# Supplementary material for: The intracellular inositol (pyro)phosphate receptor AtSPX1 reciprocally binds to P1BS DNA
Source: Nat Commun. 2026 Feb 24;17:3150. doi: 10.1038/s41467-026-69810-5 (PMC13044247; doi:10.1038/s41467-026-69810-5)
Supplement: Supplementary file 1 — Supplementary Information [file 41467_2026_69810_MOESM1_ESM.pdf]

SUPPLEMENTARY INFORMATION

The intracellular inositol (pyro)phosphate receptor AtSPX1 reciprocally binds to P1BS DNA

Whitfield, H.L.[1]*, Gilmartin, M.[1], Riley, A.M.[2], Shipton, M.L.[2], Potter, B.V.L.[2], Hemmings, A.M.[1,3] and Brearley, C.A.[1]*

[1]School of Biological Sciences, University of East Anglia, Norwich Research Park, Norwich NR4 7TJ, UK
[2]Medicinal Chemistry & Drug Discovery, Department of Pharmacology, University of Oxford, Mansfield Road, Oxford OX1 3QT, UK
[3]School of Chemistry, Pharmacy and Pharmacology, University of East Anglia, Norwich Research Park, Norwich NR4 7TJ, UK

*Email: c.brearley@uea.ac.uk
*Email: h.whitfield@uea.ac.uk

Supplementary Methods

Synthesis of 2-linked $InsP_5$ Affinity Matrix
Synthesis of 5-FAM-$InsP_5$
Protein Modelling
Molecular Dynamics Simulations and Binding Free Energy Calculations

Supplementary Figures

Supplementary Tables

Supplementary Figure 1. Synthesis of 5-FAM-$InsP_5$
Supplementary Figure 2. Binding of FAM-$InsP_5$ isomers to full-length AtSPX1
Supplementary Figure 3. The AlphaFold predicted structure of full-length AtSPX1 clashes with OsPHR2 CC domain when residues missing from the crystallised truncated SPX protein are included with published superpositions of truncated OsSPX1 and OsPHR2
Supplementary Figure 4. Visual representations of SPX1 inositol phosphate modelling values
Supplementary Figure 5. Comparison of predicted binding of inositol phosphates and diphosphoinositol phosphates as ligands of OsSPX1
Supplementary Figure 6. Calculated properties of the SPX1 complex with $InsP_6$
Supplementary Figure 7. Calculated properties of the SPX1 complex with 1,5-$[PP]_2$-$InsP_4$
Supplementary Figure 8. Calculated properties of the SPX1 complex with 3,5-$[PP]_2$-$InsP_4$
Supplementary Figure 9. Calculated properties of the SPX1 complex with 3-PP-$InsP_5$
Supplementary Figure 10. Calculated properties of the SPX1 complex with 5-PP-$InsP_5$
Supplementary Figure 11. Calculated properties of the SPX1 complex with 1-PP-$InsP_5$
Supplementary Figure 12. Calculated properties of the SPX1 complex with 5-PP-$InsP_4$
Supplementary Figure 13. Root mean square deviations of non-hydrogen atoms of ligands and binding site residues.
Supplementary Figure 14. Free energy landscapes for SPX1 and SPX1-ligand complexes
Supplementary Figure 15. DNA and InsP binding to AtSPX1 preparations

Supplementary Figure 16. Superposition of binding orientations of 4x P1BS DNA models generated by RoseTTAFoldNA
Supplementary Figure 17. Synthesis of 2-linked $InsP_5$ matrix
Supplementary Figure 18. Affinity purification of AtSPX1 on resin-bound P1BS dsDNA

Supplementary Table 1. Induced fit docking (IFD) scores and free energies (ΔG) of binding of inositol phosphates and diphosphoinositol phosphates to OsSPX. ΔG (with standard deviation) was obtained from the MM/PBSA approach.
Supplementary Table 2. Free energies (ΔG) of binding of inositol phosphates and diphosphoinositol phosphates to OsSPX
Supplementary Table 3. Computational scanning alanine mutagenesis of OsSPX1 binding site residues.
Supplementary Table 4. FAM-labelled or unlabelled primer sequences.
Supplementary Table 5. Suppressed ion conductivity HPLC of Pi content of P1BS dsDNA and Pi standards.
Supplementary Table 6. Reliability and Reproducibility Checklist for Molecular Dynamics Simulations.
Supplementary Table 7. Composition of Simulation Systems.

References

Supplementary Methods

General chemistry methods were as previously described [1].

Synthesis of 2-linked $InsP_5$ Affinity Matrix.

Coupling of 2-*O*-(2-aminoethyl)-Ins(1,3,4,5,6)P_5 (**1**) [1] (Supplementary Figure 17) with Affi-Gel 10 was carried out under non-aqueous conditions, taking advantage of the solubility of the triethylammonium salt of **1** in methanol. Trials showed that this method typically gave ≥ 2 µmole of immobilised $InsP_5$ per mL of settled gel as determined by total phosphate assay, while much lower coupling efficiencies were obtained when aqueous buffers were used. Thus, Affi-Gel 10 (Bio-Rad, UK, approximately 20 mL of slurry) was transferred to a sintered glass funnel and washed well with cold isopropyl alcohol (5 × 20 mL), leaving a moist cake of gel. The gel was then added to a solution of the triethylammonium salt of **1** (56 mg, 57 µmol, containing approximately 3.6 TEA^+ ions per molecule of **1**) in cold anhydrous methanol (10 mL) in a dry 100 mL round-bottom flask. Dry triethylamine (70 µL) was added, and the flask was then sealed and tumbled in an ice bath for 4 h, then at room temperature for 12 h. Excess ethanolamine (15 µL) was added to cleave any unreacted succinimidyl esters and tumbling was continued at room temperature for a further 4 h. The slurry was transferred to a clean sintered glass funnel and washed well with methanol, then milliQ water (5 × 20 mL of each). The gel was stored at 4 °C as a slurry in 0.9% w/v aqueous NaCl containing 0.02% w/v NaN_3 as preservative. Unreacted triethylammonium salt of **1** could be recovered by concentrating the combined washings and purifying the residue on Q Sepharose Fast Flow resin, eluting with triethylammonium bicarbonate (TEAB) buffer as previously reported [1].

Synthesis of 5-FAM-InsP$_5$

5-FAM-InsP$_5$ (**8**, Supplementary Figure 1) consists of Ins(1,3,4,5,6)P$_5$ coupled to 5-carboxyfluorescein via a linker attached to a terminal oxygen atom of the 5-phosphate group of the InsP$_5$. This isomer of InsP$_5$ is a *meso*-compound, with a plane of symmetry through C-2 and C-5 atoms. The synthesis of conjugates is therefore simplified by functionalising the InsP$_5$ at either O-2 [1] or O-5. Thus, reaction at O-5 of protected inositol **2** [2] with bifunctional phosphitylating agent **3**, followed by oxidation gave phosphate triester **4**. Compound **4** has a stereogenic centre at the 5-phosphorus atom and no longer has a plane of symmetry, existing as a racemic mixture at this stage. The two butane-2,3-diacetal (BDA) protecting groups were now removed selectively by acid hydrolysis and phosphitylation of the tetraol product **5**, followed by oxidation of phosphites, then gave fully protected **6**. The benzyl protecting groups on the phosphates and O-2 were then removed by catalytic hydrogenolysis; at this point, the *meso* symmetry was re-established. Finally, the *N*-trifluoroacetyl protecting group was cleaved by careful alkaline hydrolysis with triethylamine in water. It was possible to follow the progress of this step by obtaining successive ^{31}P NMR spectra in D_2O of samples from the reaction mixture. The product was purified by ion-exchange chromatography on Q Sepharose Fast Flow resin, eluting with aqueous TEAB to give the triethylammonium salt of **7**, containing approximately 4 triethylammonium ions per molecule of **7**. The aminopropyl-functionalised compound **7** was then reacted with an excess of 5-carboxyfluorescein succinimidyl ester in aqueous triethylammonium bicarbonate (TEAB). A ^{31}P NMR spectrum in D_2O of a sample taken from the reaction mixture showed that the reaction was approximately 80% complete after 24 h. Interestingly, this reaction was much easier than the equivalent reaction of 5-carboxyfluorescein succinimidyl ester with 2-*O*-(2-aminoethyl)-IP$_5$ (**1**) [1], presumably because the nucleophilicity of the amine group in **1** is reduced by interactions with flanking phosphate groups at C-1 and C-3 and by the short (two-carbon) alkyl chain. Purification on Q Sepharose Fast Flow resin successfully removed unreacted **7** and fluorescent byproducts, giving **8** as the triethylammonium salt. Because high concentrations of TEAB buffer (up to 2 moldm^{-3}) were required to elute **8**, the product contained cationic alkylammonium buffer residues, which were evident in ^{1}H and ^{13}C NMR spectra of **8** at this stage. These residues were removed using Chelex-100 resin (Na^+ form), followed by lyophilisation to give pure 5-FAM-InsP$_5$ as the sodium salt, which was quantified by total phosphate assay.

Benzyl [3-(2,2,2-trifluoroacetamido)propyl] diisopropylphosphoramidite (3). To a stirred solution of *N*-(3-hydroxypropyl)trifluoroacetamide [3] (860 mg, 5.0 mmol) in dry CH_2Cl_2 (10 mL) under N_2 was added benzyloxybis(diisopropylamino)phosphine [4] (2.0 g, 5.9 mmol) and 1*H*-tetrazole (300 mg, 4.28 mmol). After 3 h, the mixture was diluted with CH_2Cl_2 (80 mL), washed with aqueous TEAB (1.0 moldm^{-3}, 100 mL), dried over $MgSO_4$, and concentrated. Purification of the residue by flash chromatography (EtOAc:hexane:triethylamine, 2:8:1 then 3:7:1 gave the product as a colourless oil (1.20 g, 2.94 mmol, 59%); ^{1}H NMR (270 MHz, $CDCl_3$): δ 1.19 (d, J = 7.0 Hz, 12H), 1.85 (tt, J = 5.9 Hz, 2H), 3.49 (dt, J = 5.9 Hz, 2H), 3.58–3.67 (m, 2H), 3.71–3.88 (m, 2H), 3.60–4.80 (m, 2H), 7.24–7.35 (m, 5H); ^{31}P NMR (109 MHz, $CDCl_3$): δ 148.71.

2-*O*-Benzyl-1,6:3,4-bis-*O*-(2,3-dimethoxybutane-2,3-diyl)-*myo*-inositol 5-benzyl [3-(2,2,2-trifluoroacetamido)propyl]phosphate (4). To a stirred solution of **3** (490 mg, 1.20 mmol) in dry CH_2Cl_2 (5 mL) was added 1*H*-tetrazole (105 mg, 1.50 mmol). The mixture became thick

and cloudy within 5 min, after which time alcohol **2** [2] (499 mg, 1.00 mmol) was added. After 1.5 h, the mixture was cooled to –78 °C and *m*-chloroperoxybenzoic acid (57%, 690 mg, 2.0 mmol) was added in portions over 1 min. The mixture was allowed to reach room temperature, then diluted with CH_2Cl_2 (50 mL), washed with 10% aq. Na_2SO_3 solution (50 mL), dried over $MgSO_4$ and concentrated by evaporation under reduced pressure. The residue was purified by flash chromatography eluting with EtOAc/hexane (1:2 then 1:1) to give **4** as a white foam (740 mg, 0.900 mmol, 90%);^{1}H NMR (400 MHz, $CDCl_3$): δ 1.19 (s, 3H), 1.21 (s, 3H), 1.29 (s, 3H), 1.30 (s, 3H), 1.83 (tt, J = 5.6 Hz, 2H), 3.17 (s, 3H), 3.21 (s, 3H), 3.23 (s, 6H), 3.36–3.44 (m, 1H), 3.47–3.55 (m, 1H), 3.55–3.60 (m, 2H), 3.81 (t, J = 2.4 Hz, 1H), 4.03–4.13 (m, 1H), 4.15–4.23 (m, 3H), 4.15–4.23 (m, 3H), 4.46 (q, J = 9.6 Hz, 1H), 4.88 (broad s, 2H), 5.13–5.23 (m, 2H), 7.24–7.39 (m, 8H), 7.50–7.52 (d, J = 7.2 Hz, 2H), 8.13 (broad t, 1H); ^{13}C NMR (126 MHz, $CDCl_3$): δ 17.48, 17.59, 17.60, 28.91, 35.36, 47.80, 47.81, 47.93, 47.98, 64.05, 68.01, 68.12, 68.76, 69.28, 73.94, 75.84, 77.70, 99.27, 99.31, 99.59, 115.93, 127.08, 127.63, 127.92, 128.47, 128.56, 136.00, 139.39, 157.50; ^{31}P NMR (162 MHz, $CDCl_3$): δ 0.37; ^{19}F NMR (471 MHz, $CDCl_3$): δ 75.95; HRMS (*m/z*): $[M–OCH_3]^+$ calcd. for $C_{37}H_{51}F_3NO_{14}P$, 790.2810; found 790.2852.

2-*O*-Benzyl-*myo*-inositol 5-benzyl[3-(2,2,2-trifluoroacetamido)propyl]phosphate (5). To compound **4** (191 mg, 0.232 mmol) was added 95% aqueous trifluoroacetic acid (2 mL). The solution was stirred at room temperature for 30 min, then concentrated under reduced pressure (no heat) to give a solid residue. Purification by flash chromatography (dichloromethane/methanol 15:1) gave tetraol **5** as a white solid (84 mg, 0.142 mmol, 61%); ^{1}H NMR (270 MHz, CD_3OD): δ 1.88 (tt, J = 6.7 Hz, 2H), 3.32–3.44 (m, 2H), 3.50 (dd, J = 9.9 Hz, 2.2 Hz, 2H), 3.80–3.88 (two overlapping t, J = 9.6 Hz, 2H), 3.92 (t, J = 2.2 Hz, 1H), 4.04–4.18 (m, 3H), 4.87 (broad s, 2H), 5.17 (d, $^3J_{HP}$ = 7.4 Hz, 2H), 7.21–7.44 (m, 10H), 9.22 (broad s, 1H); ^{31}P NMR (109 MHz, CD_3OD): δ –1.01.

2-*O*-Benzyl-*myo*-inositol 5-benzyl[3-(2,2,2-trifluoroacetamido)propyl]phosphate 1,3,4,6-tetrakis(dibenzylphosphate) (6). To a suspension of 1*H*-tetrazole (80 mg 1.14 mmol) and tetraol **5** (84 mg, 0.142 mmol) in dry CH_2Cl_2 (2 mL) under N_2 was added bis(benzyloxy)diisopropylaminophosphine (256 mg, 0.682 mmol). The mixture was stirred at room temperature for 1.5 h and then cooled to –78 °C, before *m*-CPBA (57%, 345 mg, 1.14 mmol) was added. The mixture was allowed to warm to room temperature and then diluted with CH_2Cl_2 (30 mL). The clear solution was washed with 10% aq. sodium metabisulphite solution (30 mL), dried over $MgSO_4$ and concentrated by evaporation under reduced pressure. The residue was purified by flash chromatography eluting with EtOAc/hexane (1:1 to 3:1 then EtOAc) to give the title compound as a colourless oil (198 mg, 0.121 mmole, 85%); ^{1}H NMR (270 MHz, $CDCl_3$): δ 1.51–1.62 (m, 2H), 3.15–3.32 (m, 2H), 3.74–3.94 (m, 2H), 4.19–4.38 (m, 2H), 4.34 (q, J = 9.5 Hz, 1H), 4.71–5.16 (m, 22H), 7.11–7.29 (m, 45H), 8.85 (broad t, 1H); ^{31}P NMR (109 MHz, $CDCl_3$): δ –1.59 (1P), –1.25 (2P), –0.94 (1P), –0.39 (1P); MS (FAB, m/z): $[M+H]^+$ calcd for $C_{81}H_{83}F_3NO_{22}P_5$ 1634.41, found 1634.4.

***Myo*-inositol 5-(3-aminopropylphosphate) 1,3,4,6-tetrakisphosphate (7).** To a solution of **6** (190 mg, 116 *m*mol) in MeOH (32 mL) and water (8 mL) was added Pd–C (10 %, 50% water, 400 mg). The mixture was shaken in a Parr hydrogenator under H_2 (50 p.s.i.) for 24 h. The catalyst was removed by filtration through a PTFE syringe filter and triethylammonium bicarbonate solution 1.0 M TEAB (1 mL) was added. The solvents were removed by evaporation under reduced pressure and methanol was repeatedly added and evaporated,

eventually leaving a colourless glassy residue (approx. 140 mg) Analysis of this material by ^{1}H and ^{31}P NMR spectroscopy showed that all benzyl protecting groups had been removed. The residue was re-dissolved in in water (10 mL) and triethylamine (1 mL) was added. The solution was stirred at room temperature for 18 h and then concentrated. The product was purified by ion-exchange chromatography on Q-Sepharose Fast Flow resin eluting with a gradient of TEAB (0 to 2.0 moldm^{-3}) to give the pure triethylammonium salt of **7** as a colourless glass (101 mg, 97 µmole, 84% yield over two steps); ^{1}H NMR (270 MHz, D_2O): δ 1.22 (t, J = 7.4 Hz, approx. 36H), 1.98 (tt, J = 5.9 Hz, 2H), approx. 3.1 (buried, 2H), 3.13 (q, J = 7.4 Hz, approx. 24H), 4.06–4.22 (m, 5H), 4.31 (broad s, 1H), 4.49 (q, J = 9.4 Hz, 2H); ^{31}P NMR (109 MHz, D_2O): δ 0.65 (3P), 0.99 (2P); HRMS (*m/z*): M$^-$ calcd. for $C_9H_{24}NO_{21}P_5$ 635.9444; found 635.9451.

***Myo*-inositol 5-[3-(5-fluoresceinylcarboxy)aminopropylphosphate] 1,3,4,6-tetrakisphosphate (5-FAM-InsP$_5$, 8).** To a stirred solution of **7** (15 mg of 4TEA$^+$ salt, 14 µmol) in deionised water (750 µL) was added TEAB (1.0 moldm^{-3}, 250 µL) followed by 5-carboxyfluorescein NHS ester [5] (28 mg, 59 mmole) in DMF (500 µL). The flask was covered in foil to exclude light. The reaction mixture was stirred at room temperature for 24 h and then concentrated under reduced pressure. The residue was dissolved in TEAB (0.05 moldm^{-3}, pH approx. 7.5, 50 mL) and applied to a small column of Q Sepharose Fast Flow resin (bicarbonate form, 70 mm x 20 mm). The column was washed well with milliQ water followed by TEAB (0.8 moldm^{-3}, pH approx. 7.8) until the eluent ran colourless. The column was then eluted with a gradient of TEAB (0.8 moldm^{-3} to 2.0 moldm^{-3}) over 300 mL, collecting 10 mL fractions. A fluorescent product eluted at high buffer concentration (> 1.6 moldm^{-3} TEAB). Fractions containing this product were combined and concentrated to give an orange solid. The solid was re-dissolved in milliQ water (5 mL) and filtered through a column of Chelex-100 resin (Na$^+$ form, 5 cm deep in a Pasteur pipette), which was then washed with further milliQ water (5 mL). The combined eluents were lyophilised to give the sodium salt of **8** as a fluffy orange powder (13 mg), which was accurately quantified by total phosphate assay (yield 8.7 µmole, 62 %); ^{1}H NMR (270 MHz, D_2O) δ 1.98 (tt, J approx. 7 Hz, 2H), 3.53 (t, J = 7.2 Hz, 2H), 4.01–4.23 (m, 5H), 4.40 (q, *J* = 9.4, 4.42, 2H), 4.42 (broad s, 1H), 6.56–6.61 (m, 4H), 7.15 (d, J = 9.6 Hz 2H), 7.41 (d, J = 8.0 Hz, 1H), 7.96 (dd, J = 8.0, 1.9 Hz, 1H), 8.14 (d, J = 1.9 Hz, 1H); ^{31}P NMR (109 MHz, D_2O, triethylamine added): δ 0.19 (1P), 3.14 (2P), 4.31 (2P); HRMS (m/z): M$^-$ calcd. for $C_{30}H_{34}NO_{27}P_5$, 993.9934; found 993.9945; Analytical RP-HPLC: R_t = 4.71 min. The chromatographic system consisted of a Phenomenex Security Guard cartridge system for HPLC and a Phenomenex Gemini 5 mm C_{18} 10 Å column (150 x 4.6 mm), eluted at 1 mLmin^{-1} with a gradient of 5% to 70% of acetonitrile in 0.1 moldm^{-3} aqueous triethylammonium acetate over 10 min, with detection at 254 nm.

Protein Modelling

Induced Fit Docking (IFD) was performed using the IFD module of the Maestro-Schrödinger suite (Schrödinger Release 2020-3: Induced Fit Docking protocol; Glide, Schrödinger, LLC, New York, NY, 2020; Prime, Schrödinger, LLC, New York, NY, 2020). Starting ligand coordinates were generated from Ligand Expo [6]. Initial Glide docking for each ligand was carried out based on a docking box the size of which was generated automatically for each ligand. Residue side chains were trimmed based on their B-factors and van der Waals scale factors of 0.7 and 0.5 were utilised for non-polar atoms of the protein and ligand, respectively. The limiting number of poses generated was set to 20. Prime side-chain

prediction and minimization were then carried out in which residues were refined within 6.0 Å of ligand poses and side chain conformations were optimised. The refined complexes were arranged by prime energy and, finally, Glide extended precision (XP) redocking was carried out into structures within 30.0 kcal/mol of the best structure, and within the top 20 structures overall. In this way, the ligand was rigorously redocked into the induced-fit receptor structure and the results yielded an IFD score in kcal mol^{-1} for each output pose. The more negative the IFD score the higher the binding affinity. The visualization of the best poses of each of the predicted protein-ligand complexes was performed using PyMOL (The PyMOL Molecular Graphics System, Version 2.5 Schrödinger, LLC).

Molecular Dynamics Simulations and Binding Free Energy Calculations

Subsequently, ten independent molecular dynamics (MD) trajectories were calculated over 10 ns for each of the SPX1-ligand complexes immersed in an explicit TIP3P water bath. The resulting trajectories were used to calculate ligand binding free energies by the MM/PBSA (Molecular Mechanics/Poisson-Boltzmann Surface Area) method.

Briefly, the starting ligand and binding site structure for each was based on the atomic coordinates resulting from induced fit docking in Schrodinger (see Protein Modelling section above). Forcefield parameters for the ligands were prepared using ACPYPE (AnteChamber PYthon Parser interface) [7] with partial atomic charges assigned using the AM1-BCC method [8]. During the preparation of the starting structures for simulation, a short region of unresolved polypeptide in the crystal structure of rice SPX1 was modelled using the corresponding region of entry Q69XJ0 from the AlphaFold Protein Structure Database [9,10]. The protonation state of protein residues for each complex in the presence of ligand was predicted with H++ [11] and used during the MD simulations. The topology and coordinate files for the protein were generated using pdb2gmx from GROMACS taking parameters from the Amber99SB-ILDN force field [12]. The coordinate and topology files of the protein and the ligands were then merged to obtain the final starting structure and topology file for each complex.

Molecular dynamics (MD) simulations were performed using GROMACS version 2025.2 [13]. The complex was placed in a dodecahedral periodic box and solvated by the addition of TIP3P water molecules [14]. The minimum distance between any atom in a solute and the periodic box edge was set to 10 Å. The net charge on the system was then neutralised by adding counterions as required and the ionic strength adjusted by the addition NaCl to a concentration of 150 mM. Nonbonded interactions were truncated at 10 Å and the particle mesh Ewald (PME) was used to treat long-range electrostatic interactions [15,16]. System temperature was controlled with a Langevin thermostat with a collision frequency 2.0. The energy of each simulation system was first minimised using 5000 iterations of the steepest descent method with constraints on all protein and ligand non-hydrogen atoms (restraining force constant 1000 kJ/mol/nm^2). To equilibrate the explicit water molecules in the simulation, the system was then heated to 300 K and evolved over 200 ps at constant volume with 1 fs time step. The pressure was then equilibrated to 1 atm during a 500 ps NPT simulation. In both simulations, all non-hydrogen atoms of the protein and ligand were position-restrained with a force constant of 1000 kJ/mol/nm^2. The position restraints on the ligand were then gradually relaxed over a further two 500 ps NPT simulations to yield a final restraining force constant of 1 kJ/mol/nm^2 over non-hydrogen atoms of the protein

mainchain and ligand. In these pressure equilibration steps both temperature and pressure were regulated using the Berendsen algorithm [17].

Production simulations were performed for 10 ns with a 1 fs time step. The temperature and pressure were maintained at 300 K and 1 atm using the v-rescale temperature [18] and Berendsen pressure coupling method. The time constant for the temperature and pressure coupling was kept at 0.1 and 1 ps, respectively. The short-range nonbonded interactions were computed for the atom pairs within the cutoff of 1 nm, while the long-range electrostatic interactions were calculated using particle-mesh-Ewald summation method with fourth-order cubic interpolation and 1.2 Å grid spacing [15]. All bonds were constrained using the parallel LINCS method [19, 20]. Protein non-hydrogen main chain atoms were restrained with force constant 1 kJ/mol/nm^2 to avoid unnecessary structural drift due to inaccuracy of the force field [21, 22]. No restraints were applied to atoms of the ligands. The coordinates of the system were saved every 50 fs for post simulation calculations. This process was repeated to generate a total of 10 independent trajectories for each ligand starting from its energy minimized structure by use of a random number seed to allocate initial atomic velocities. The number of replicate simulations chosen follows the recommendations of Knapp et al.[23] Monitoring root mean square deviation, root mean square fluctuation, surface accessible area and the number of hydrogen bonds involving the ligands allowed us to monitor the onset of equilibration which was achieved for all trajectories and ligands by 5 ns (see Supplementary Figures 6-13). Clustering of conformations of non-hydrogen ligand and binding site atoms (including residues 1-5, 25-29 and 147-151) over the time period 5-10 ns for all independent replicate trajectories yielded representative structures of the bound ligands. The middle structure of the most populous cluster was chosen as representative of the bound state of that ligand i.e., that pose with the lowest average RMSD to all other structures within the cluster (Supplementary Figure 4). Free Energy Landscape analysis was performed for each of the ligands bound to OsSPX1 and for the uncomplexed protein using the sham module of GROMACS [24].

The GROMACS output trajectories for each ligand were fitted and periodic boundary conditions removed before running energy calculations with gmx_MMPBSA v1.6.4 [25]. A total of 100 frames from the last 5 ns of each trajectory (i.e. frames every 50 ps) and thus 1000 frames in total were used to calculate the binding free energy, as suggested elsewhere [26]. The individual binding free energy values can be found in Supplementary Table 2. The same Amber99SB-ILDN force field as was used to generate the molecular trajectories was used to calculate the internal term as well as van der Waals and electrostatic energies. The non-polar desolvation energy was calculated using default parameters. The same selection of frames was used in a computational alanine scanning mutagenesis [27,28] experiment performed for eight binding site residues (Met1, Lys2, Lys5, Tyr25, Lys29, Lys149, Lys150 and Lys151), with a specific internal dielectric constant as suggested [29] and chosen according to the physicochemical properties of the mutated amino acid.

Literature precedent suggests that the accuracy of the chosen models is sufficient to address the question under investigation [30,31].

Supplementary Figures

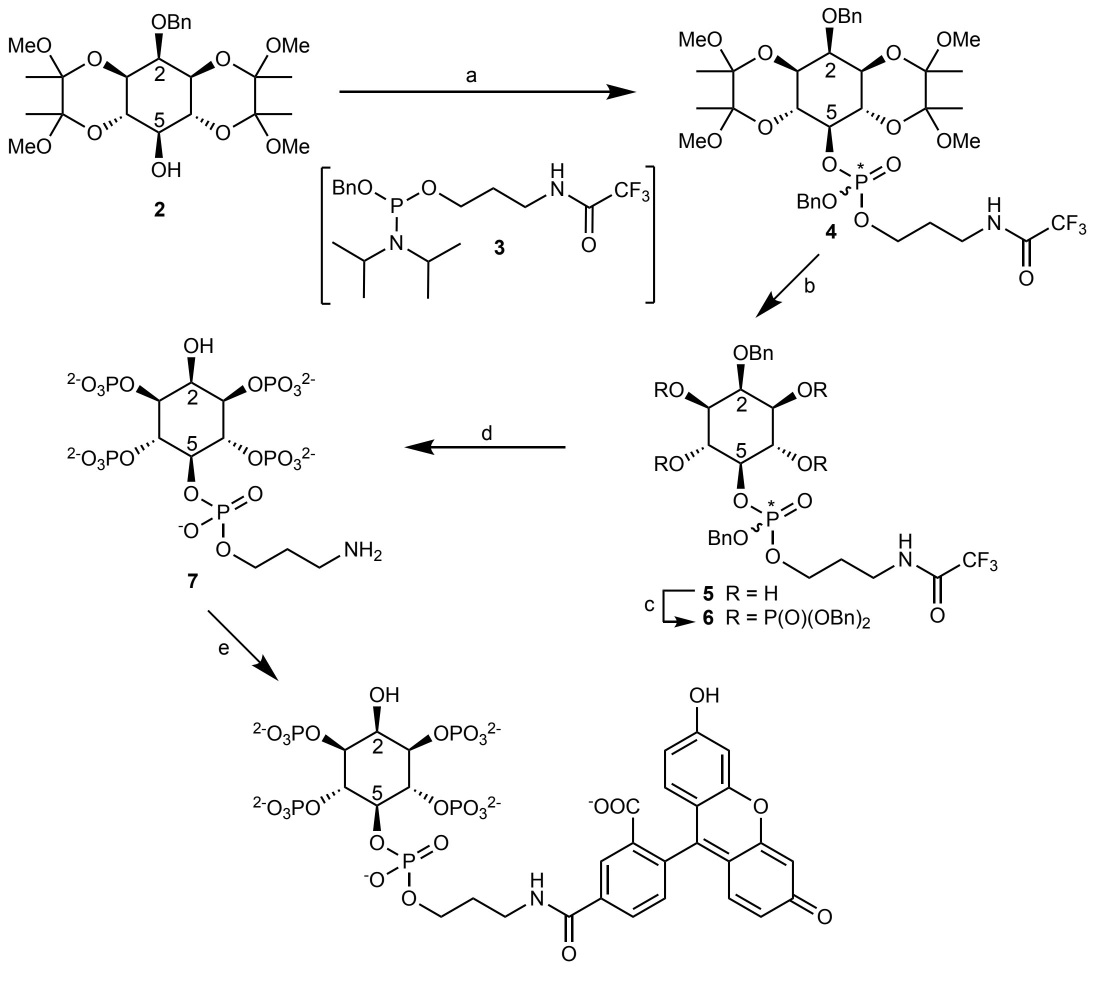

Supplementary Figure 1. Synthesis of 5-FAM-InsP$_5$. Reagents and conditions: a) i. **3**, 1*H*-tetrazole, CH_2Cl_2; ii. *m*-CPBA, –78 °C, 90%; b) TFA, H_2O, 61%; c) i. $(BnO)_2PNPr^i_2$, 1*H*-tetrazole, CH_2Cl_2; ii. *m*-CPBA, –78 °C, 85%; d) i. H_2, Pd-C, MeOH, H_2O, 50 p.s.i.; ii. triethylamine, H_2O, 84%; e) 5-carboxyfluorescein succinimidyl ester, DMF, H_2O TEAB, 62%. Bn = benzyl, TEAB = triethylammonium bicarbonate, * = stereogenic centre.

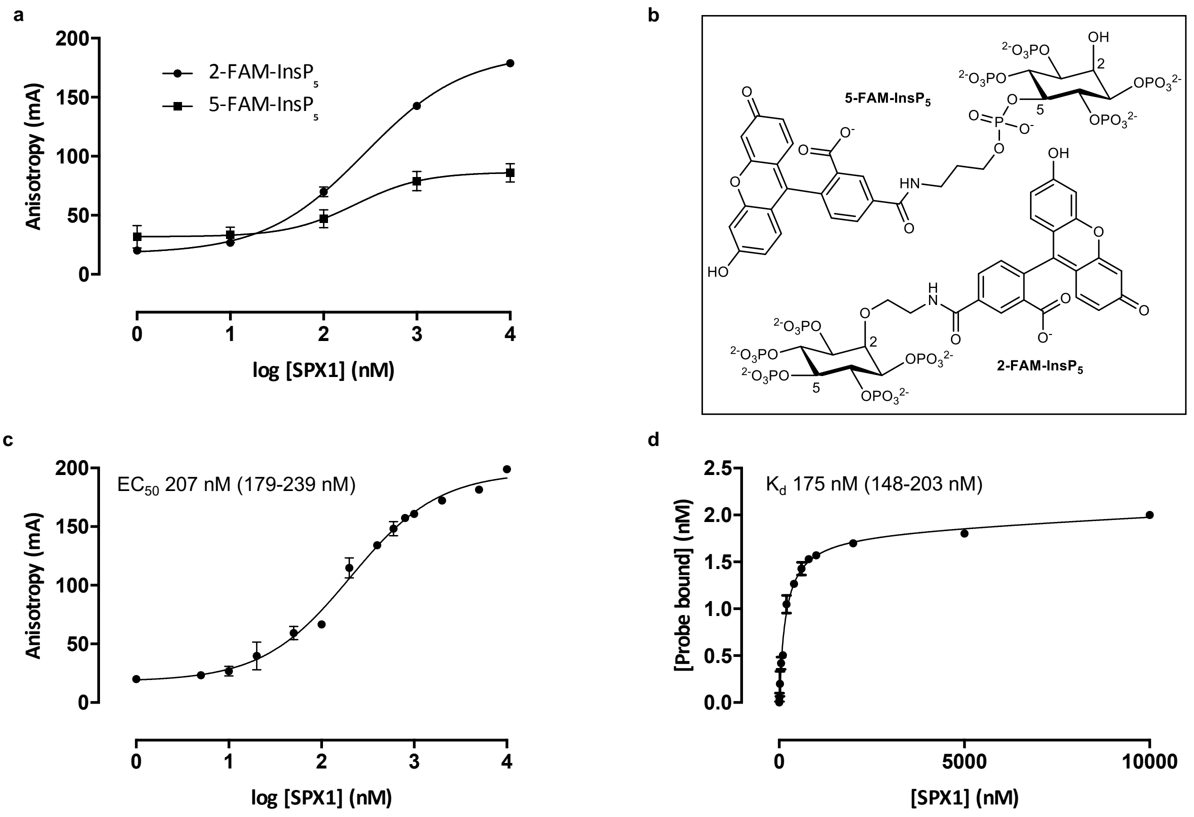

Supplementary Figure 2. Binding of FAM-InsP$_5$ isomers to full-length AtSPX1. (a) Comparisons of binding isotherms for 2-FAM-InsP$_5$ and 5-FAM-InsP$_5$. Probes at 2nM concentration were incubated in 20 mM HEPES pH 6.5, 1 mM $MgCl_2$, 100 mM NaCl with increasing concentration of full-length SPX1. N= 4 replicates (b) Chemical structures of 5-FAM-InsP$_5$ and 2-FAM-InsP$_5$. (c) Detailed binding isotherm for 2-FAM-InsP$_5$ N= 4 replicates (d) Data transformed into fraction bound, fitted to a one site - total binding model. K_d shown with 95% confidence interval, N= 4 replicates.

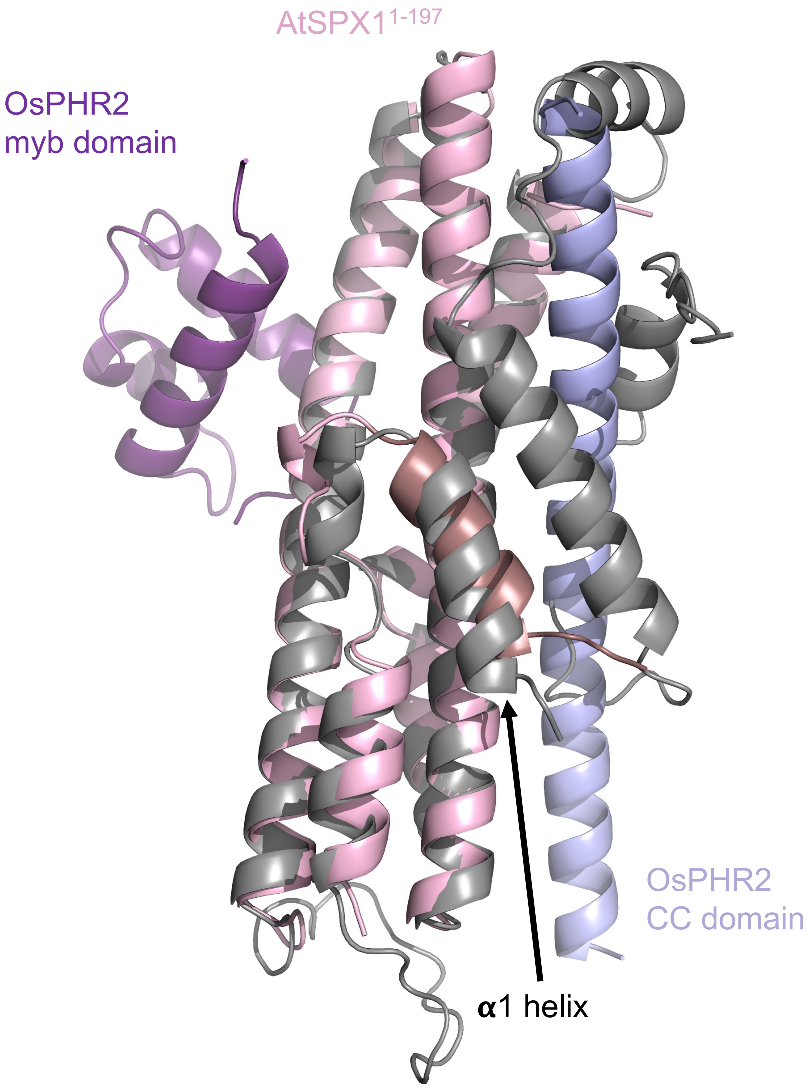

Supplementary Figure 3. The AlphaFold [32] predicted structure of full-length AtSPX1 clashes with OsPHR2 CC domain when residues missing from the crystallised truncated SPX protein are included with published superpositions of truncated OsSPX1 and OsPHR2. Rice OsSPX1 structure (PDB: 7E40) [7] of AtSPX$^{1-198}$ (pink), OsPHR2 CC domain (blue), OsPHR1 myb domain (purple) superposed with the AlphaFold model of full length AtSPX1 (grey).

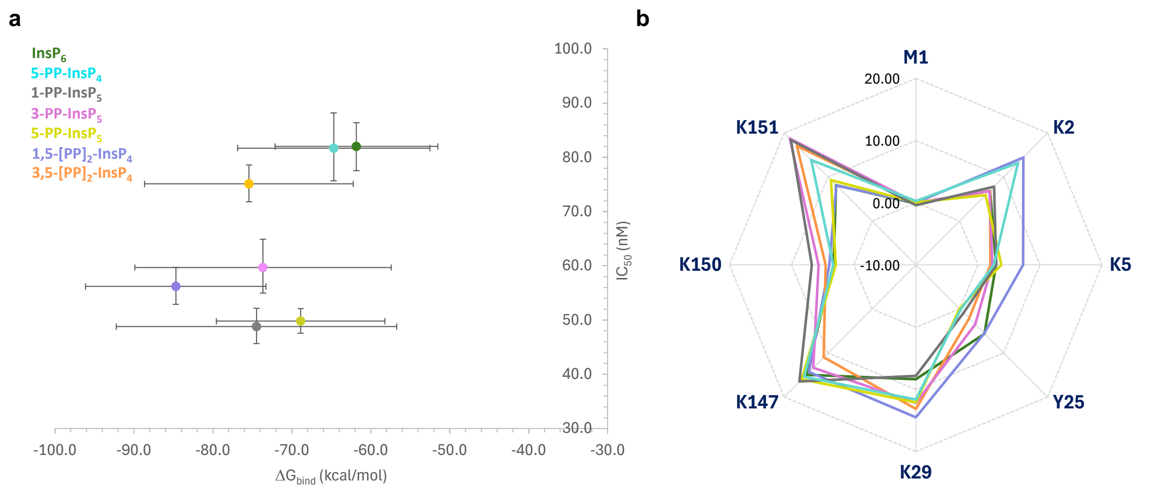

Supplementary Figure 4. Visual representations of SPX1-inositol phosphate modelling values (a) correlation between IC_{50} values (with 95% confidence interval) as calculated in Figure 1a and b and ΔG_{bind} estimates (with SD) generated from molecular dynamics simulations of each ligand (for numerical values see Supplementary Table 2); (b) A spider graph showing the results of computational scanning alanine mutagenesis of selected binding site residues for each of seven inositol phosphate and diphosphoinositol phosphate ligands. Energy units are kJ/mol. For numerical values see Supplementary Table 3.

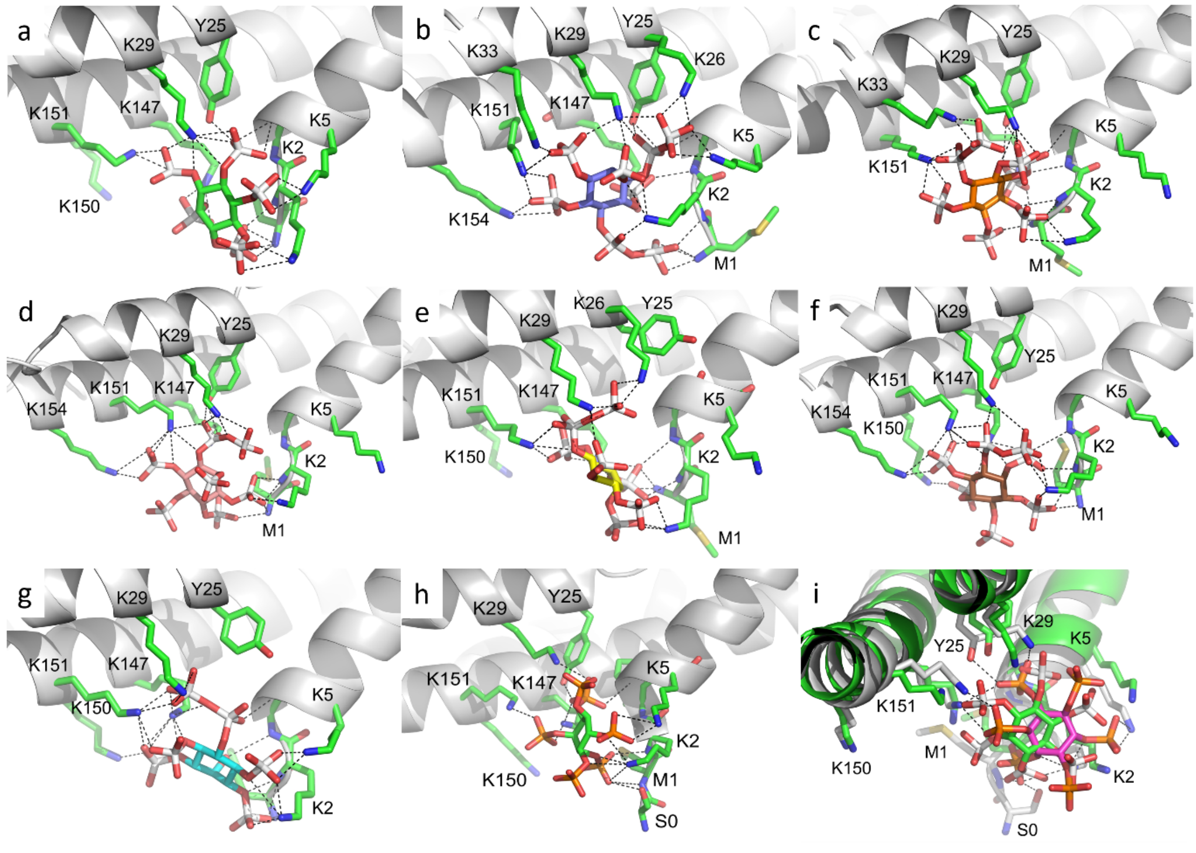

Supplementary Figure 5. Comparison of predicted binding of inositol phosphates and diphosphoinositol phosphates as ligands of OsSPX1. (a) $InsP_6$; (b) 1,5-$[PP]_2$-$InsP_4$; (c) 3,5-$[PP]_2$-$InsP_4$; (d) 3-PP-$InsP_5$; (e) 5-PP-$InsP_5$; (f) 1-PP-$InsP_5$; (g) 5-PP-$InsP_4$ and (h) $InsP_6$ bound to OsSPX1 from PDB entry 7E40. (i) superposition of predicted (green carbon atoms, green cartoon) and crystallographic (PDB 7E40, $InsP_6$ with magenta carbon atoms and OsSPX1 with grey atoms and grey cartoon) poses of $InsP_6$ bound to OsSPX1. Polar interactions are indicated by black dashed lines. Individual residues predicted to interact with the ligands are shown as sticks and are labelled. Note that hydrogen atoms, water molecules and counter ions have been removed for clarity.

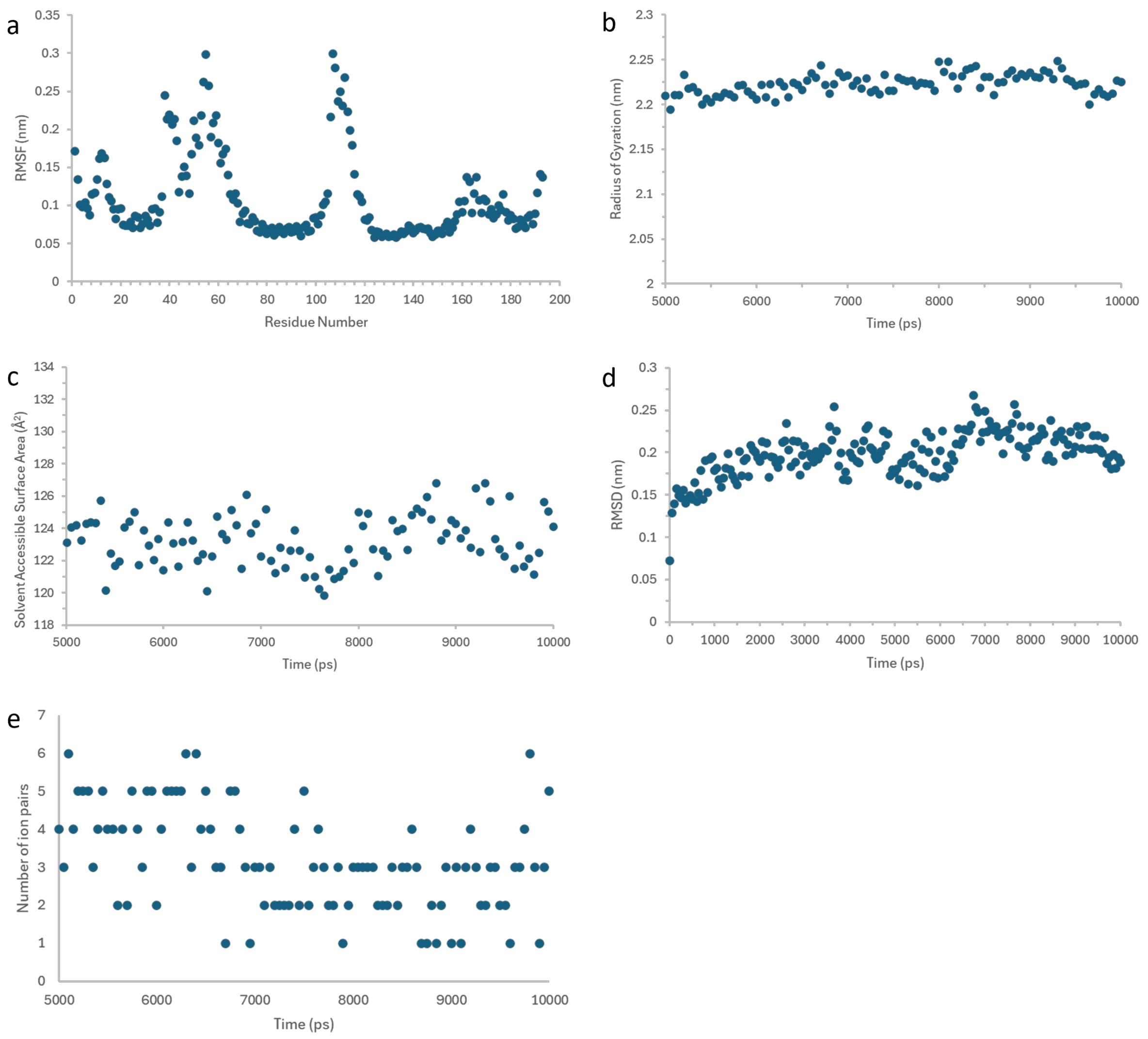

Supplementary Figure 6. Calculated properties of the SPX1 complex with $InsP_6$ during the production phase of a typical molecular dynamics trajectory. (a) Root mean square fluctuations; (b) Radius of gyration; (c) Solvent accessible surface area; (d) Evolution of root mean square deviation from initial model coordinates; (e) Number of ion pair interactions with the ligand. Note that properties in panels (a), (b) and (d) have been calculated using the coordinates of main chain atoms.

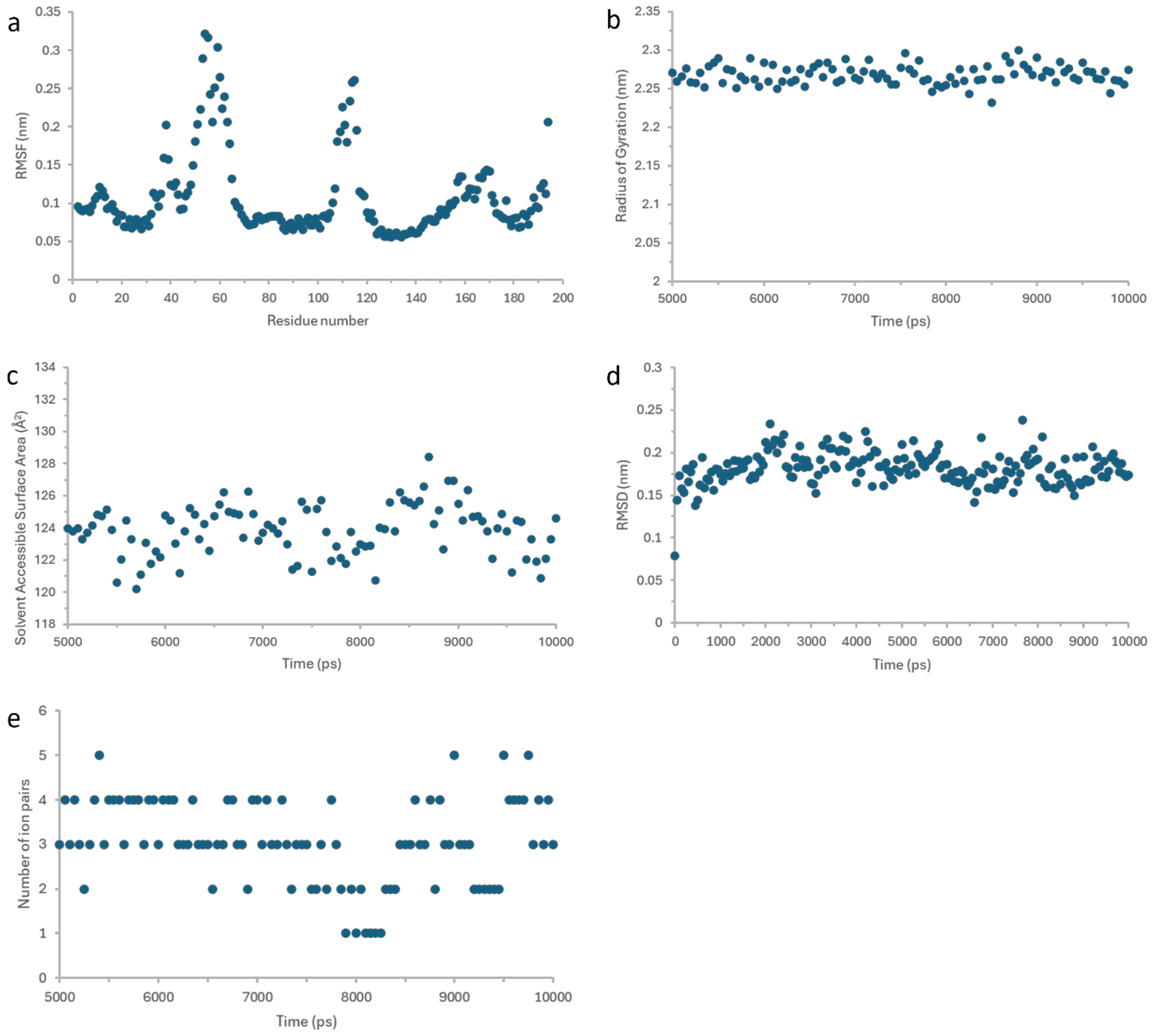

Supplementary Figure 7. Calculated properties of the SPX1 complex with 1,5-[PP]$_2$-InsP$_4$ during the production phase of a typical molecular dynamics trajectory. (a) Root mean square fluctuations; (b) Radius of gyration; (c) Solvent accessible surface area; (d) Evolution of root mean square deviation from initial model coordinates; (e) Number of ion pair interactions with the ligand. Note that properties in panels (a), (b) and (d) have been calculated using the coordinates of main chain atoms.

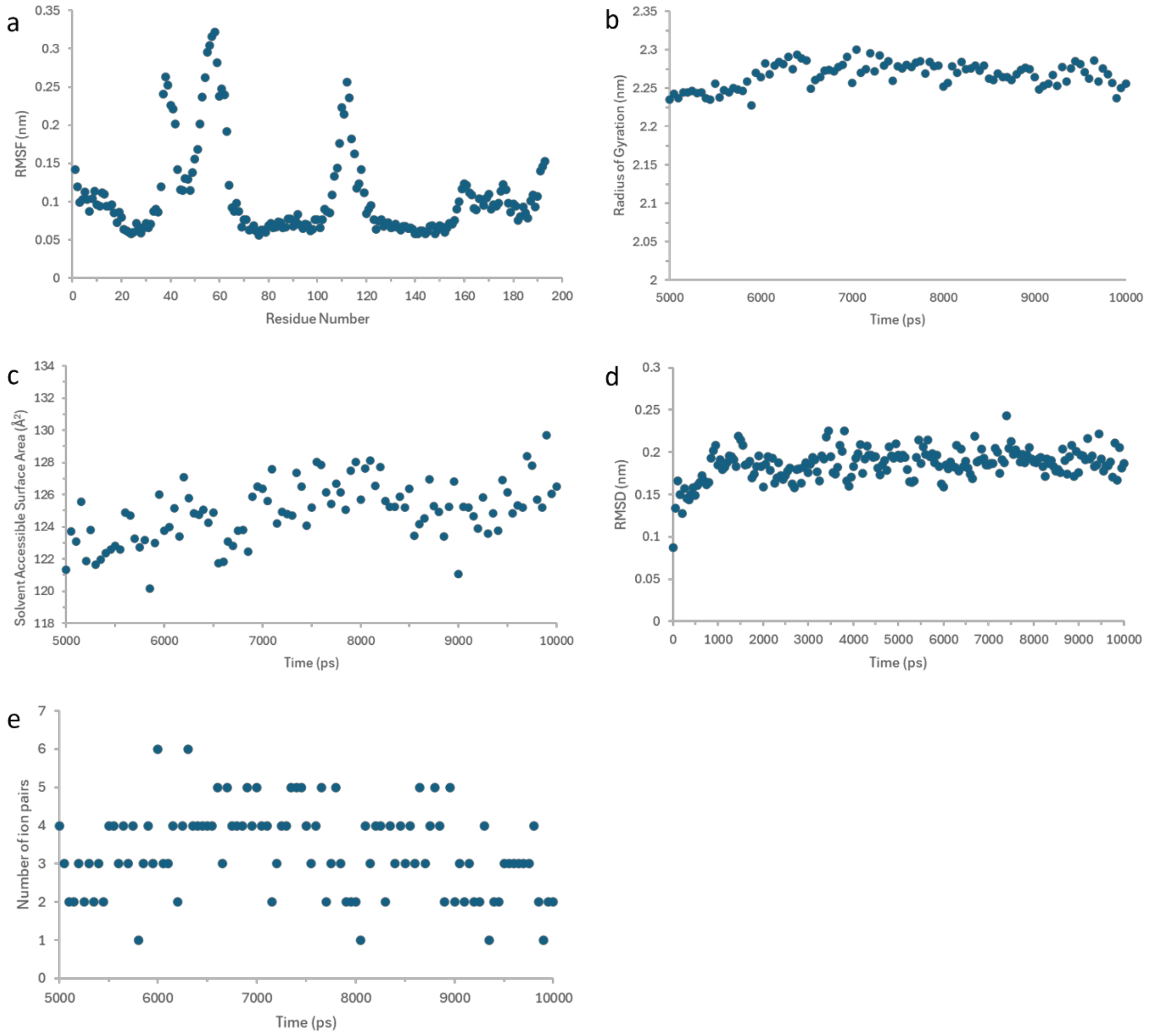

Supplementary Figure 8. Calculated properties of the SPX1 complex with 3,5-[PP]$_2$-InsP$_4$ during the production phase of a typical molecular dynamics trajectory. (a) Root mean square fluctuations; (b) Radius of gyration; (c) Solvent accessible surface area; (d) Evolution of root mean square deviation from initial model coordinates; (e) Number of ion pair interactions with the ligand. Note that properties in panels (a), (b) and (d) have been calculated using the coordinates of main chain atoms.

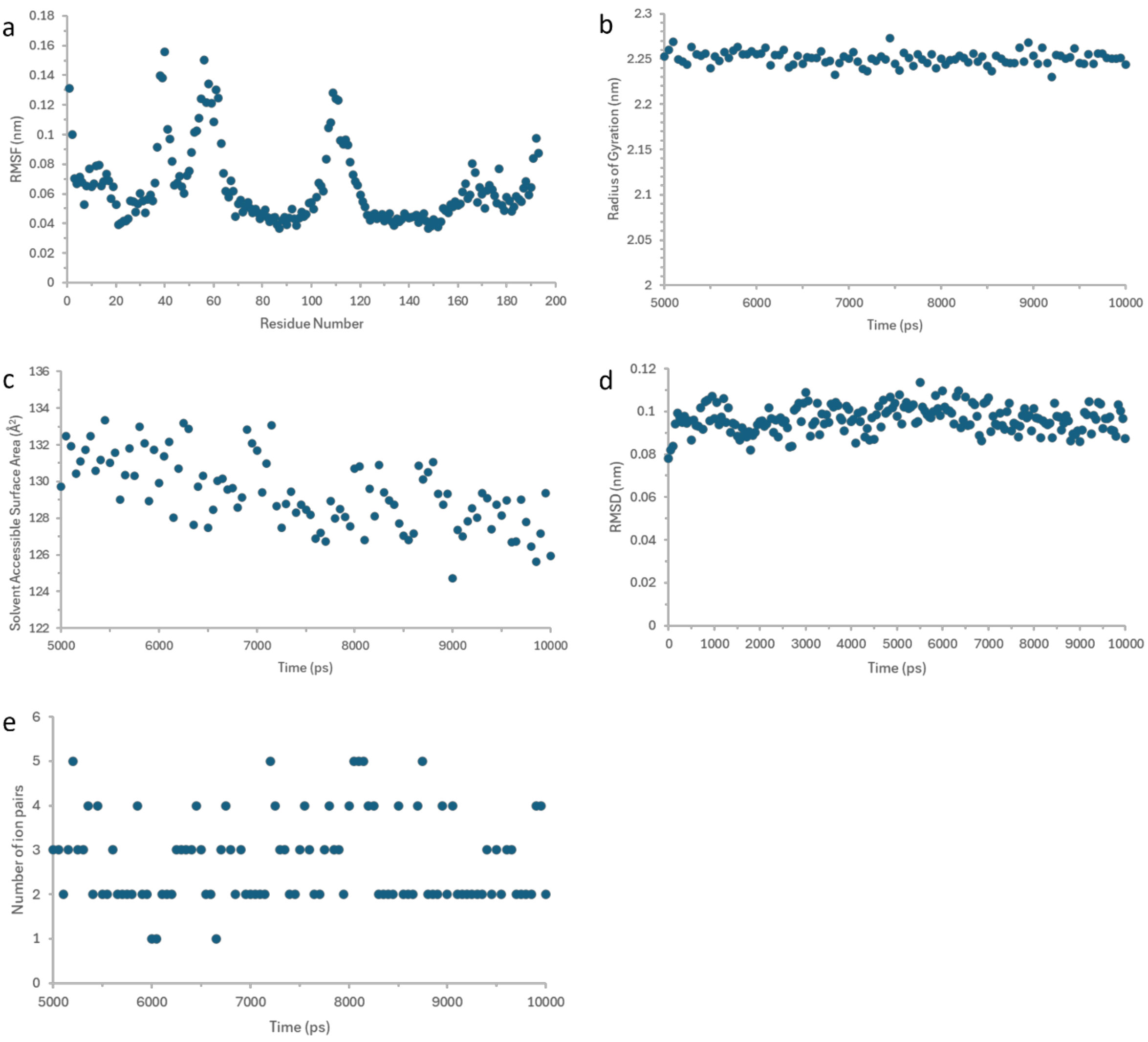

Supplementary Figure 9. Calculated properties of the SPX1 complex with 3-PP-InsP$_5$ during the production phase of a typical molecular dynamics trajectory. (a) Root mean square fluctuations; (b) Radius of gyration; (c) Solvent accessible surface area; (d) Evolution of root mean square deviation from initial model coordinates; (e) Number of ion pair interactions with the ligand. Note that properties in panels (a), (b) and (d) have been calculated using the coordinates of main chain atoms.

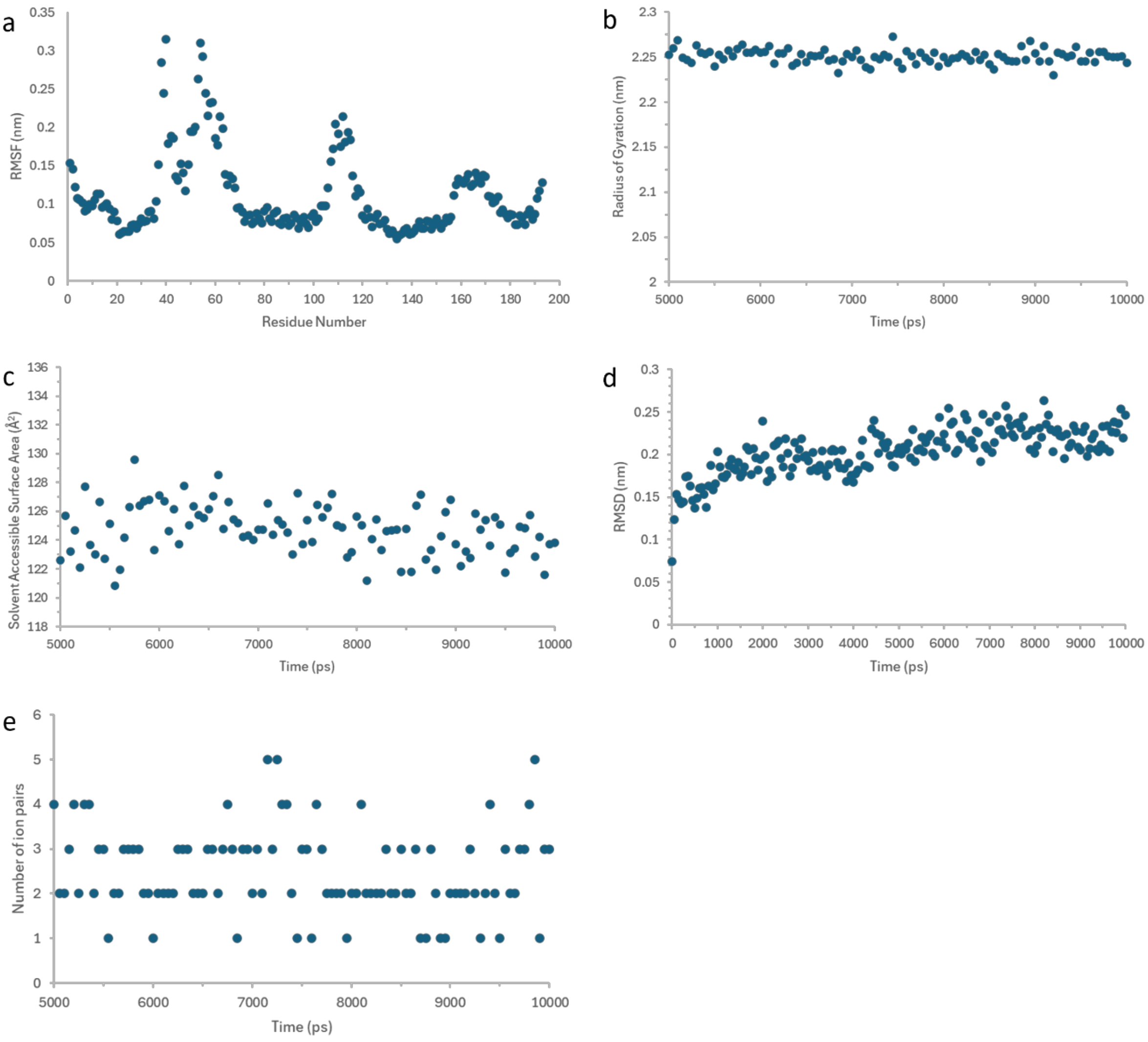

Supplementary Figure 10. Calculated properties of the SPX1 complex with 5-PP-InsP$_5$ during the production phase of a typical molecular dynamics trajectory. (a) Root mean square fluctuations; (b) Radius of gyration; (c) Solvent accessible surface area; (d) Evolution of root mean square deviation from initial model coordinates; (e) Number of ion pair interactions with the ligand. Note that properties in panels (a), (b) and (d) have been calculated using the coordinates of main chain atoms.

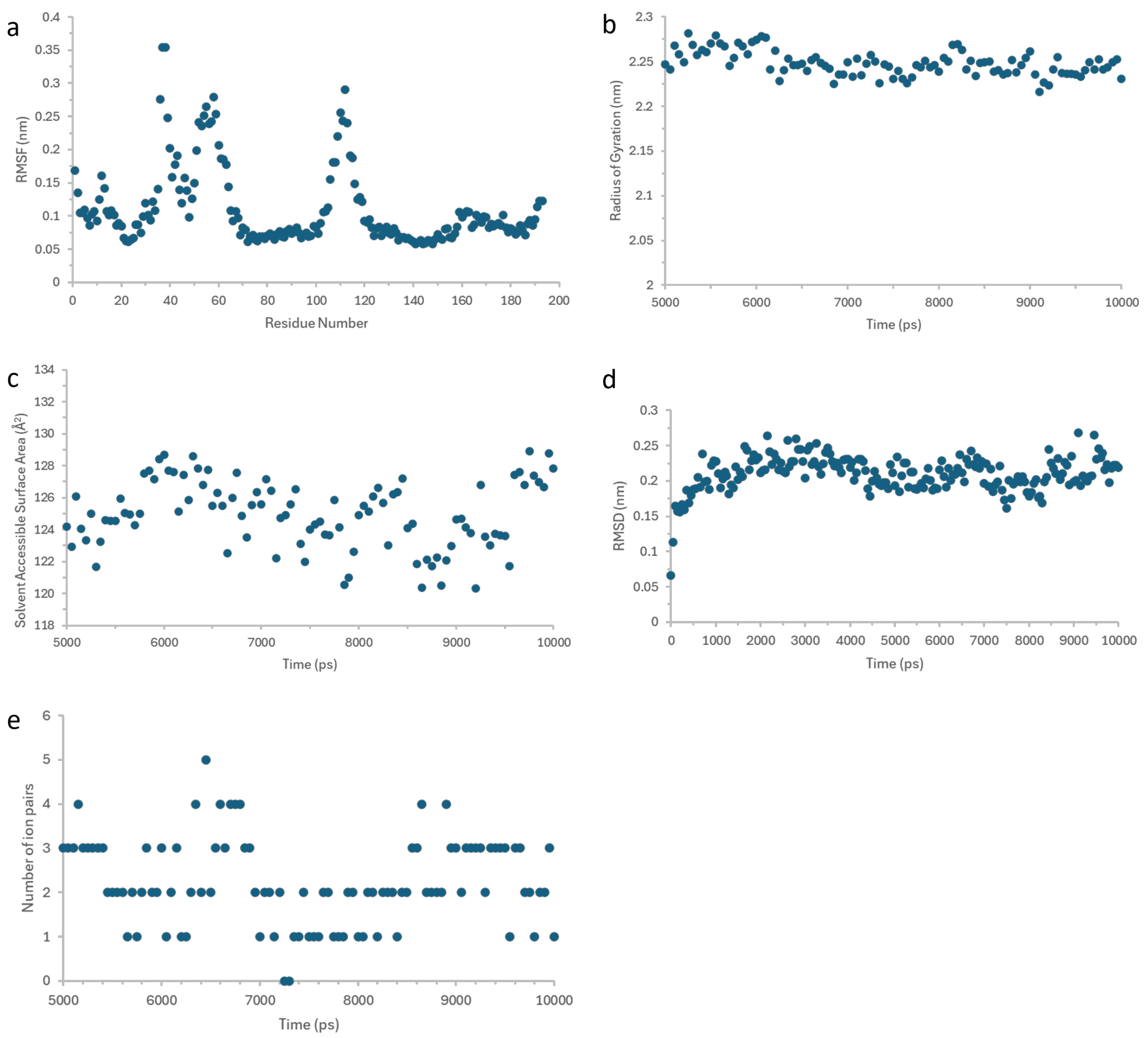

Supplementary Figure 11. Calculated properties of the SPX1 complex with 1-PP-InsP$_5$ during a typical molecular dynamics trajectory. (a) Root mean square fluctuations; (b) Radius of gyration; (c) Solvent accessible surface area; (d) Evolution of root mean square deviation from initial model coordinates; (e) Number of ion pair interactions with the ligand. Note that properties in panels (a), (b) and (d) have been calculated using the coordinates of main chain atoms.

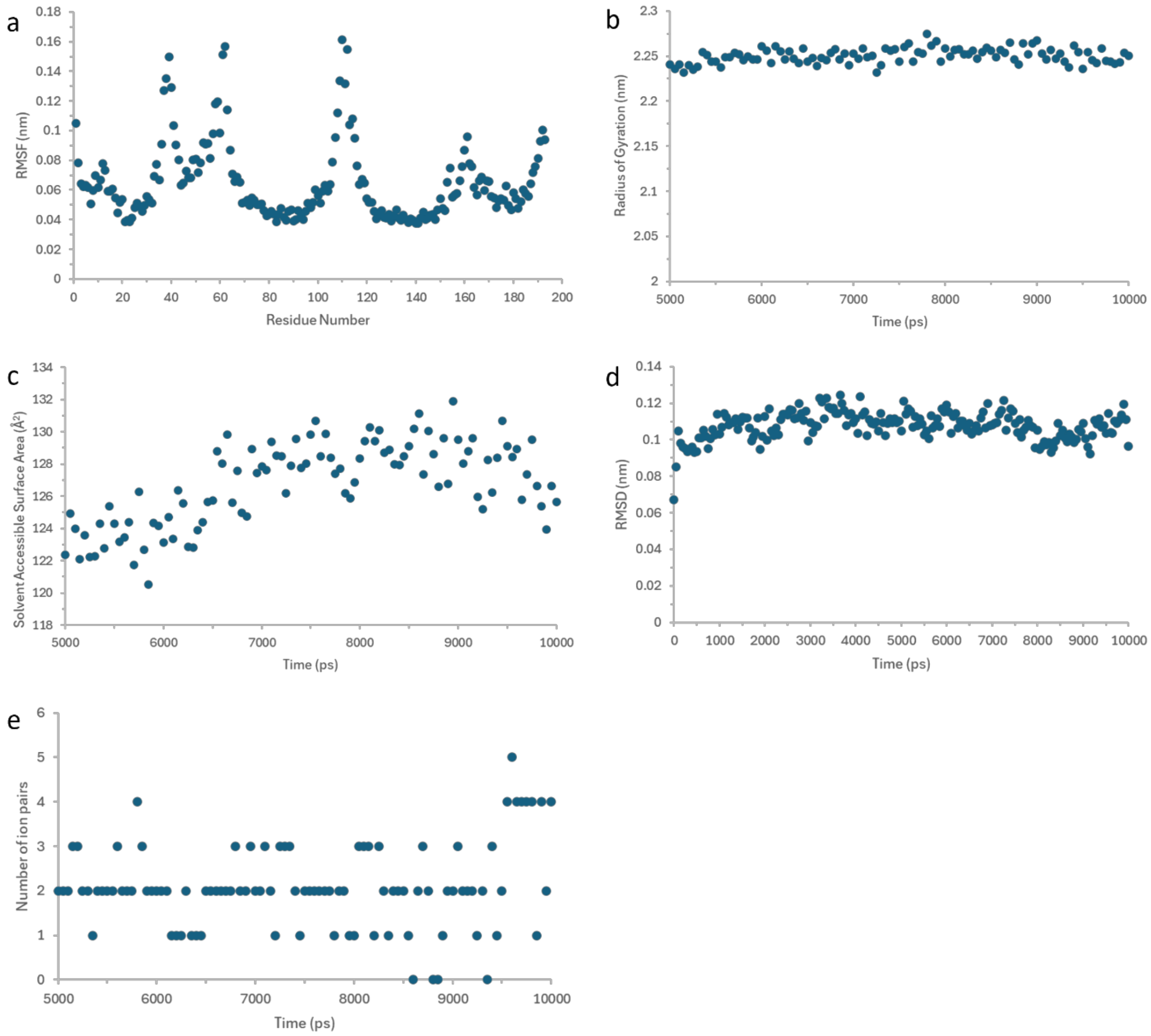

Supplementary Figure 12. Calculated properties of the SPX1 complex with 5-PP-InsP$_4$ during the production phase of a typical molecular dynamics trajectory. (a) Root mean square fluctuations; (b) Radius of gyration; (c) Solvent accessible surface area; (d) Evolution of root mean square deviation from initial model coordinates; (e) Number of ion pair interactions with the ligand. Note that properties in panels (a), (b) and (d) have been calculated using the coordinates of main chain atoms.

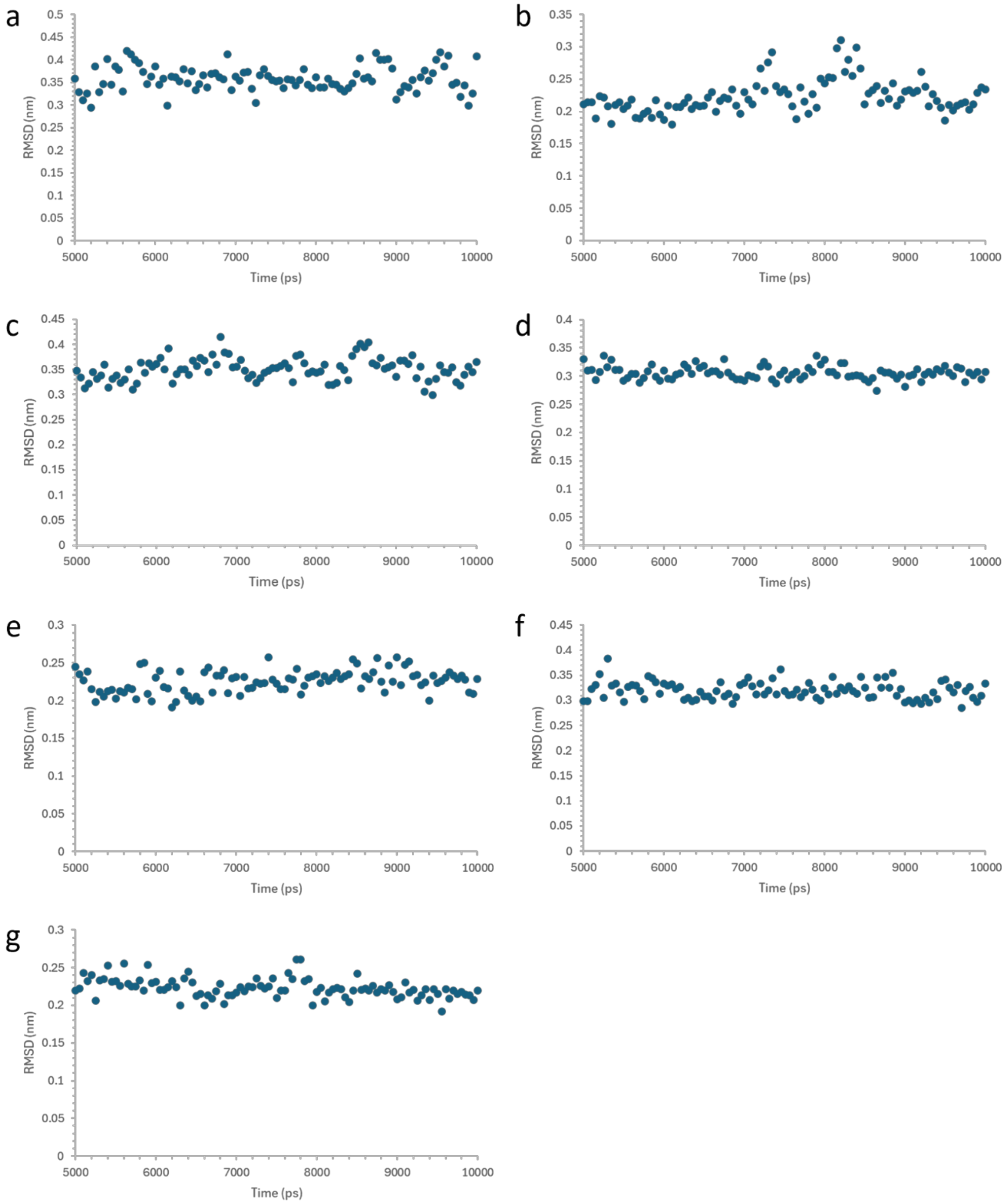

Supplementary Figure 13. Root mean square deviations of non-hydrogen atoms of ligands and binding site residues (M1, K2, K5, Y25, K29, K147, K150 and K151) from their initial positions over the production phase of molecular dynamics simulations. RMSD values are shown at 50 ps intervals at which binding free energy estimates were calculated. (a) $InsP_6$; (b) 1,5-$[PP]_2$-$InsP_4$; (c) 3,5-$[PP]_2$-$InsP_4$; (d) 3-PP-$InsP_5$; (e) 5-PP-$InsP_5$; (f) 1-PP-$InsP_5$; (g) 5-PP-$InsP_4$

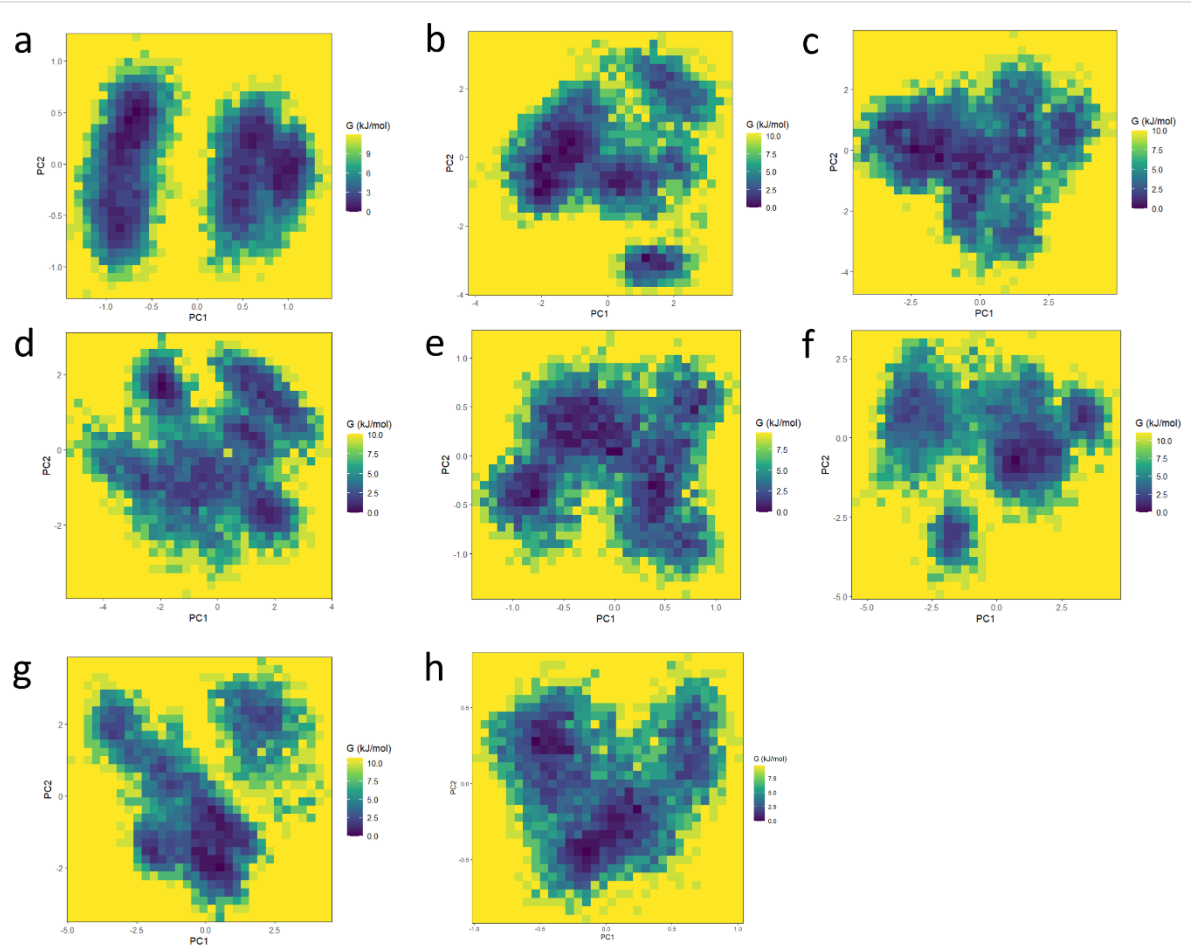

Supplementary Figure 14. Free energy landscapes for SPX1 and SPX1-ligand complexes. Surfaces were calculated using frames sampled at 50 ps intervals over 10 independent simulations each of 5 ns duration for (a) the uncomplexed OsSPX1 protein and the protein in complex with (b) $InsP_6$; (c) 1,5-$[PP]_2$-$InsP_4$; (d) 3,5-$[PP]_2$-$InsP_4$; (e) 3-PP-$InsP_5$; (f) 5-PP-$InsP_5$; (g) 1-PP-$InsP_5$; (h) 5-PP-$InsP_4$. Plots are shown for the first two principal components calculated from all backbone atom positions. A graduated colour scheme showing free energy in $kJmol^{-1}$ is shown to the right of each plot.

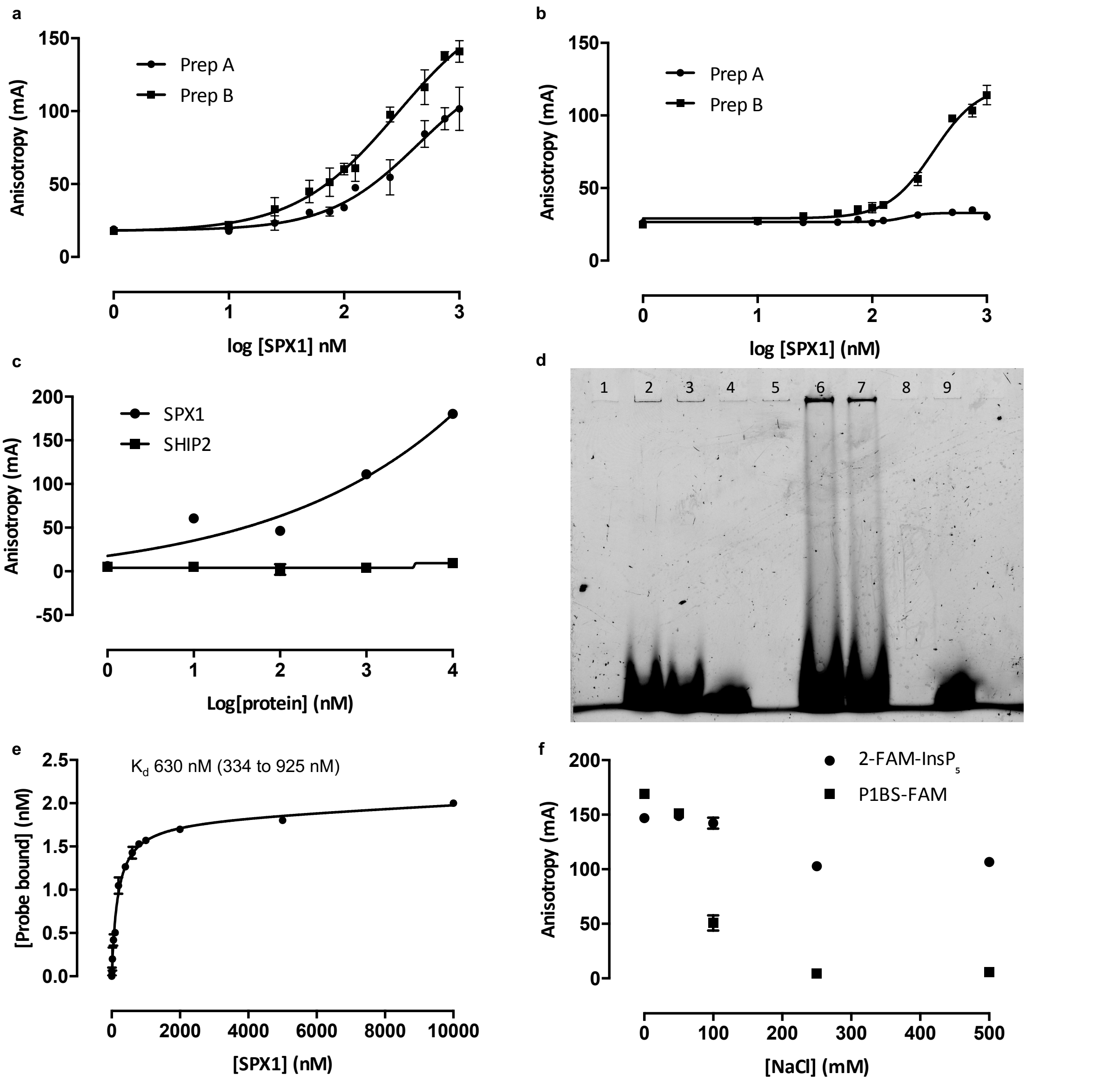

Supplementary Figure 15. DNA and InsP binding to AtSPX1 preparations. (a) Anisotropy of 2-FAM-InsP$_5$ probe incubated with increasing concentration of either Prep A (high A$_{260:280}$ ratio) or B (low A$_{260:280}$ ratio) AtSPX1. N= 4 replicates. (b) Anisotropy of 5′-FAM-12mer DNA probe incubated with increasing concentration of either Prep A or B AtSPX1. N= 4 replicates. (c) Anisotropy of 5′-12mer FAM-DNA probe incubated with increasing concentration of either AtSPX1 or HsSHIP2. N= 4 replicates. (d) PAGE of 12mer DNA binding to AtSPX1. Lanes: 1, Prep A + 100 mM NaCl; 2, Prep A + DNA; 3, Prep A + 100 mM NaCl + DNA; 4, 100 mM NaCl + DNA; 5, Prep B + 100mM NaCl; 6, Prep B + DNA; 7, Prep B + 100 mM NaCl + DNA; 8, AtITPK4 + 100 mM NaCl; 9, AtITPK4 + 100 mM NaCl + DNA. Retention of DNA/protein in well (at top of gel) is reminiscent of EMSA (on agarose) of PHR2$^{180\text{-}313}$: P1BS (Supplementary Figure 11, of reference [33]). Repeated twice with similar results. (e) 5′ -FAM-P1BS bound concentration against increasing AtSPX1 concentration, allowing calculation of K_d with its 95% confidence interval (completed under identical conditions to that of 2-FAM-InsP$_5$ data shown in Supplementary Figure 2c, d). N= 4 replicates. (f) Effect on anisotropy of increasing NaCl concentration on full length AtSPX1 binding to 2-FAM-InsP$_5$ compared to FAM-P1BS. N= 4 replicates.

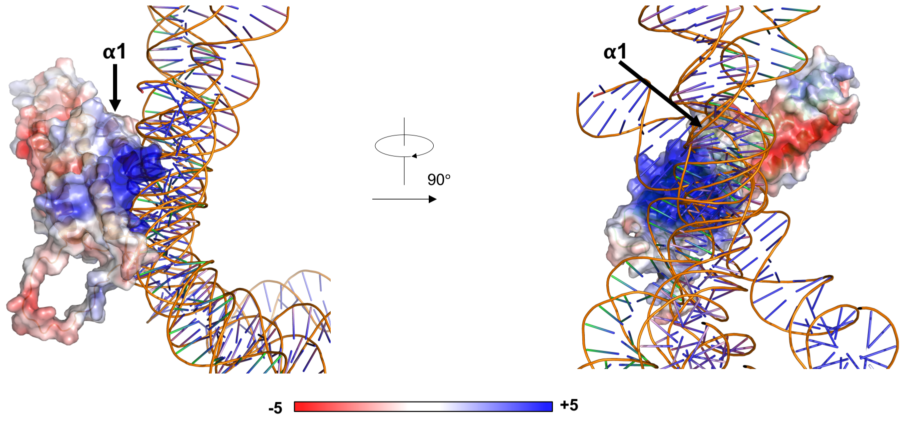

Supplementary Figure 16. Superposition of binding orientations of 4x P1BS DNA models generated by RoseTTAFoldNA [34] using OsSPX1 full length, OsSPX1$^{1\text{-}198}$, AtSPX1 full length and AtSPX1$^{1\text{-}198}$ as receptors. For simplicity, only the OsSPX1$^{1\text{-}198}$ receptor is shown, coloured according to electrostatic charge.

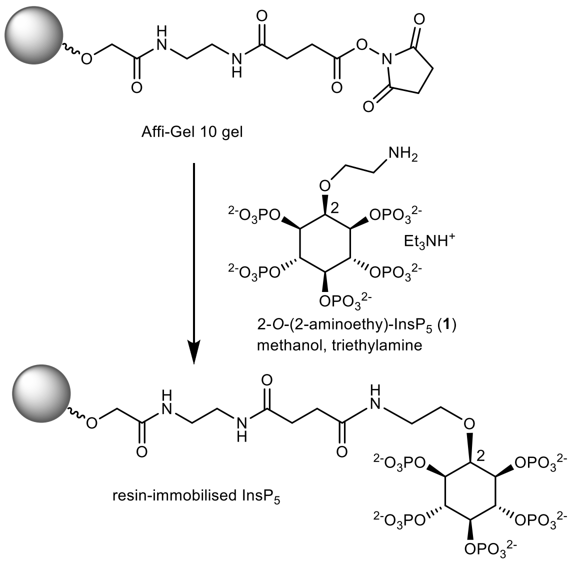

Supplementary Figure 17. Synthesis of 2-linked $InsP_5$ matrix.

	2	3	4	5	6	7	8
P1BS tagged beads	-	-	+	-	+	+	-
Control beads	-	+	-	+	-	-	+
AtSPX1	+	+	+	+	+	+	+
$InsP_6$	-	-	-	-	-	-	-

	2	3	4	5	6	7	8
P1BS tagged beads	-	+	+	-	+	+	-
Control beads	-	-	-	+	-	-	+
AtSPX1	+	+	+	+	+	+	+
$InsP_6$	-	+	-	+	+	-	+

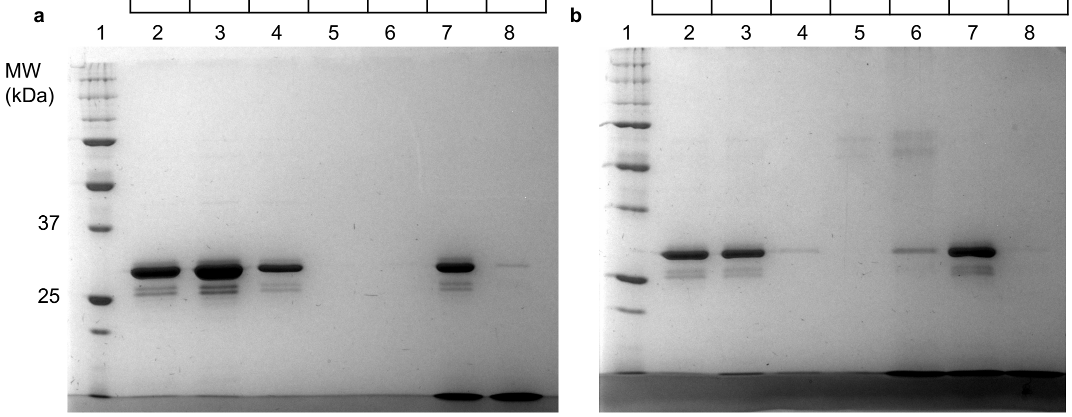

Supplementary Figure 18. Affinity purification of AtSPX1 on resin-bound P1BS dsDNA. Biotinylated P1BS-DNA was immobilised on Streptavidin Sepharose and the resin-conjugate loaded with AtSPX1. Protein fractions, eluting or not, were analysed by SDS PAGE. (a) Lanes: 1, Ladder; 2 AtSPX1 loaded protein sample; 3, Supernatant after incubation of AtSPX1 with control beads (no DNA); 4, Supernatant after incubation of AtSPX1 with P1BS dsDNA bound beads; 5, Supernatant after washing of beads in lane 3; 6, Supernatant after washing of beads in lane 4; 7, Washed beads from lane 4 boiled in SDS buffer; 8, Washed beads from lane 3 boiled in SDS buffer. Repeated twice with similar results. (b) Lanes: 1, Ladder; 2, AtSPX1 loaded protein sample; 3, Supernatant after incubation of AtSPX1 with P1BS dsDNA bound beads in the presence of $InsP_6$; 4, Supernatant after incubation of AtSPX1 with P1BS dsDNA bound beads in the presence of water (control); 5, Supernatant after incubation of AtSPX1 with control beads (no DNA) in the presence of $InsP_6$; 6, Washed beads from lane 3 boiled in SDS buffer; 7, Washed beads from lane 4 boiled in SDS buffer; 8 Washed beads from lane 5 boiled in SDS buffer. Repeated twice with similar results.

Supplementary Tables

Supplementary Table 1. Induced fit docking (IFD) scores and free energies (ΔG) of binding of inositol phosphates and diphosphoinositol phosphates to OsSPX. ΔG (with standard deviation) was obtained from the MM/PBSA approach.

Ligand	IFD score (kcal/mol)	ΔG_{bind} (kcal/mol)
**1,5-[PP]$_2$-InsP$_4$**	-391.1	-84.7 ± 11.4
**3,5-[PP]$_2$-InsP$_4$**	-390.9	-75.4 ± 13.2
**5-PP-InsP$_5$**	-390.6	-68.9 ± 10.7
**3-PP-InsP$_5$**	-388.7	-73.7 ± 16.2
**1-PP-InsP$_5$**	-385.9	-74.5 ± 17.8
**5-PP-InsP$_4$**	-384.1	-64.7 ± 12.2
**InsP$_6$**	-376.9	-61.8 ± 10.3

Analysis of ΔG by one-way ANOVA revealed a significant difference between InsP$_6$ and 1,5-[PP]$_2$-InsP$_4$ and between 1,5-[PP]$_2$-InsP$_4$ and 3-[PP]-InsP$_5$, and between 1,5-[PP]$_2$-InsP$_4$ and 5-[PP]-InsP$_4$. Significance values can be found in source data.

Supplementary Table 2. Free energies (ΔG) of binding of inositol phosphates and diphosphoinositol phosphates to OsSPX. Individual ΔG_{bind} values calculated using the mm/PBSA method over 10 molecular dynamics trajectories each of 100 frames (5 ns per trajectory), are reported with standard deviation, SD (units of kcal/mol).

Trajectory		$InsP_6$	1,5-$[PP]_2$-$InsP_4$	3,5-$[PP]_2$-$InsP_4$	3-PP-$InsP_5$	5-PP-$InsP_5$	1-PP-$InsP_5$	5-PP-$InsP_4$
1	*Δ*Gbind	-52.18	-86.20	-71.58	-77.02	-68.00	-79.55	-58.54
	SD	5.88	6.53	10.36	8.96	10.27	9.44	4.87
2	*Δ*Gbind	-66.99	-90.38	-72.02	-70.52	-69.57	-99.86	-46.72
	SD	6.78	10.62	7.81	15.38	8.02	12.58	7.49
3	*Δ*Gbind	-68.72	-91.70	-83.00	-62.54	-76.53	-83.96	-67.32
	SD	7.33	8.55	11.85	8.60	6.73	19.98	11.42
4	*Δ*Gbind	-46.23	-87.23	-71.98	-72.37	-79.95	-88.79	-56.50
	SD	6.36	6.51	6.66	9.66	6.42	6.17	6.46
5	*Δ*Gbind	-62.02	-79.88	-74.84	-110.84	-63.96	-65.74	-62.78
	SD	8.07	8.41	9.36	11.76	7.41	13.38	8.05
6	*Δ*Gbind	-59.73	-86.54	-78.09	-69.55	-75.34	-54.12	-79.69
	SD	6.14	5.31	7.39	5.53	8.03	8.30	6.00
7	*Δ*Gbind	-58.24	-88.38	-62.30	-64.36	-61.17	-65.45	-76.67
	SD	9.86	5.49	13.24	6.74	6.67	9.79	5.51
8	*Δ*Gbind	-68.47	-87.26	-66.59	-74.96	-71.74	-72.06	-68.54
	SD	7.55	11.43	8.47	5.19	7.64	10.01	7.24
9	*Δ*Gbind	-68.14	-62.60	-98.65	-70.35	-67.80	-71.82	-72.97
	SD	7.44	10.52	7.22	7.51	8.90	7.32	8.68
10	*Δ*Gbind	-67.49	-86.77	-75.25	-64.09	-54.81	-63.45	-57.25
	SD	5.65	6.30	8.12	11.14	8.47	17.66	5.57
Overall	*Δ*Gbind	-61.80	-84.70	-75.40	-73.70	-68.90	-74.50	-64.70
	SD	10.30	11.40	13.20	16.20	10.70	17.80	12.20

Supplementary Table 3. Computational Scanning Alanine mutagenesis of OsSPX1 binding site residues. $\Delta\Delta G_{bind}$ values, calculated over 10 molecular dynamics trajectories each of 100 frames (5 ns), are reported with standard deviation (units of kcal/mol).

Mutation	$InsP_6$	1,5-$[PP]_2$-$InsP_4$	3,5-$[PP]_2$-$InsP_4$	3-PP-$InsP_5$	5-PP-$InsP_5$	1-PP-$InsP_5$	5-PP-$InsP_4$
M1	-0.12 ± 0.62	-0.01 ± 0.87	-0.03 ± 0.66	0.00 ± 1.27	0.06 ± 0.39	-0.41 ± 1.03	0.27 ± 0.72
K2	6.87 ± 4.15	14.45 ± 3.88*	6.88 ± 4.53	6.78 ± 4.01	5.87 ± 5.13	7.79 ± 3.79	13.24 ± 6.14*
K5	2.89 ± 2.35	7.28 ± 4.25*	1.98 ± 2.21	2.32 ± 0.80	3.77 ± 2.98	2.95 ± 2.18	2.55 ± 1.89
Y25	5.64 ± 5.42	5.59 ± 2.47	2.14 ± 6.10*	3.49 ± 6.42	-0.05 ± 2.60*	0.89 ± 6.81*	0.18 ± 1.70*
K29	8.38 ± 4.87	14.50 ± 3.76*	13.12 ± 5.62*	12.18 ± 7.48*	12.11 ± 3.48*	7.83 ± 6.13	11.65 ± 5.59*
K147	14.96 ± 8.87	14.31 ± 3.47	11.00 ± 9.50*	13.38 ± 9.01	15.99 ± 4.73	16.52 ± 10.09	15.41 ± 6.50
K150	3.24 ± 1.86	3.93 ± 1.84	4.51 ± 3.09	5.66 ± 4.42	2.89 ± 0.83	6.76 ± 5.12*	3.29 ± 2.04
K151	8.18 ± 3.89	8.10 ± 4.90	17.06 ± 6.15*	18.71 ± 8.44*	9.32 ± 4.29	18.45 ± 10.05*	13.84 ± 9.29*

Analysis of ΔΔG by two-way ANOVA revealed the differences between means to be significant at $P < 0.05$ by Tukey's multiple comparisons test for 72% of the pairwise comparisons. Only M1 has no significant differential effect on ΔΔG in comparisons of $InsP_6$ to each ligand. Significant differences in comparisons within mutations of the different ligands to $InsP_6$ is denoted by an asterisk. Significance values provided in source data.

Supplementary Table 4. FAM-labelled or unlabelled primer sequences.

Oligomer	Nucleotide sequence (5′ to 3′)
P1BS	GAATTGAATATGCAATG
P1BSr	CATTGCATATTCAATTC
4x P1BS	GAATTGAATATGCAATGGAATATGCTTAGGCATATTCCATAGAATATTCCTAGA
4x P1BS r	ATTAAGGAATCTATTAAGTATGAAGTGGAATGTACGCCCATTAATTGAAACTTC
12mer	CGAATTAATTCG

Supplementary Table 5. **Suppressed ion conductivity HPLC of Pi content of P1BS dsDNA and Pi standards.** Integrated peak areas for injection of 10 μl aliquots.

Sample	Area (μS.min)
100 μM Pi	0.995
10 μM Pi	0.063
1 μM Pi	0
500 nM P1BS dsDNA	0
5 μM P1BS dsDNA	0

Supplementary Table 6. Reliability and Reproducibility Checklist for Molecular Dynamics Simulations.

**1. Convergence of simulations and analysis**
1a. Is an evaluation presented in the text to show that the property being measured has equilibrated in the simulations (e.g., time-course analysis)?
Yes, see Supplementary Information Figures 6-13.
1b. Then, is it described in the text how simulations are split into equilibration and production runs and how much data were analyzed from production runs?
Yes, see Supplementary Information Methods text.
1c. Are there at least 3 simulations per simulation condition with statistical analysis?
Yes, 10 independent simulations were performed for each ligand. See Supplementary Information Methods text.
1d. Is evidence provided in the text that the simulation results presented are independent of initial configuration?
Yes, see Supplementary Information Methods text.
**2. Connection to experiments**
2a. Are calculations provided that can connect to experiments (e.g., loss or gain in function from mutagenesis, binding assays, NMR chemical shifts, J-couplings, SAXS curves, interaction distances or FRET distances, structure factors, diffusion coefficients, bulk modulus and other mechanical properties, etc.)?
Yes, indirectly. Free energies of binding should correlate with measured IC_{50} values.
**3. Method choice**
3a. Do simulations contain membranes, membrane proteins, intrinsically disordered proteins, glycans, nucleic acids, polymers, or cryptic ligand binding?
No.
3b. Is it described in the text whether the accuracy of the chosen model(s) is sufficient to address the question(s) under investigation (e.g., all-atom vs. coarse-grained models, fixed charge vs. polarizable force fields, implicit vs. explicit solvent or membrane, specific force field and water model, etc.?
Yes, see Supplementary Information Figures 6-13
3c. Is the timescale of the event(s) under investigation beyond the brute-force MD simulation timescale in this study that enhanced sampling methods are needed?
No.
If **YES**, are the parameters and convergence criteria for the enhanced sampling method clearly stated?
N/A
If **NO**, is the evidence provided in the text?
See Supplementary Information Methods text.
**4. Code and reproducibility**
4a. Is a table provided describing the system setup that includes simulation box dimensions, total number of atoms, number of water molecules, salt concentration, lipid composition (number of molecules and type)?
Yes, See Supplementary Information Table 7
4b. Are other parameters for the system setup described in the text, such as protonation state, type of structural restraints if applied, nonbonded cutoff, thermostat and barostat, etc.?
Yes. See Supplementary Information Methods text for details.
4c. Is it described in the text what simulation and analysis software and which versions are used?
Yes, see Supplementary Information Methods text.
4d. Are initial coordinate and simulation input files and a coordinate file of the final output provided as supplementary files or in a public repository?
Yes, this data is publicly available in MDRepo (https://mdrepo.org/) as entries MDR00004441, MDR00004443, MDR00004444, MDR00004445, MDR00004446, MDR00004447 and MDR00004448
4e. Is there custom code or custom force field parameters?
No.
If **YES**, are they provided as supplementary files or in a public repository?
N/A

Supplementary Table 7. Composition of Simulation Systems.

**Ligand Code**	**IP6**	**IP8**	**2YN**	**3P5**	**17P**	**O81**	**U6J**
Simulation box volume (nm^3)	888.5	888.5	888.5	888.5	888.5	888.5	888.5
Total number of atoms	87376	87401	87410	87402	87387	87405	87403
Number of residues	194	194	194	194	194	194	194
Number of water molecules	27995	28000	28003	28002	27997	28003	28004
Salt concentration (mM)	150	150	150	150	150	150	150
Number of ions (Na+, Cl-)	164	166	166	165	165	165	164
Lipid atoms	0	0	0	0	0	0	0

Code- IP6: $InsP_6$, IP8: 1,5-$[PP]_2$-$InsP_4$, 2YN: 3,5-$[PP]_2$-$InsP_4$, 3P5: 3-PP-$InsP_5$, I7P: 5-PP-$InsP_5$, O81: 1-PP-$InsP_5$, U6J: 5-PP-$InsP_4$.

References

1. Riley AM, Windhorst S, Lin HY, Potter BVL. Cellular internalisation of an inositol phosphate visualised by using fluorescent $InsP_5$. *Chembiochem* **15**, 57-67 (2014). DOI: 10.1002/cbic.201300583

2. Riley AM, Wang H, Weaver JD, Shears SB, Potter BVL. First synthetic analogues of diphosphoinositol polyphosphates: interaction with PP-$InsP_5$ kinase. *Chem Commun (Camb)* **48**, 11292-11294 (2012). DOI: 10.1039/c2cc36044f

3. Franzini RM, Kool ET. Efficient nucleic acid detection by templated reductive quencher release. *J Am Chem Soc* **131**, 16021-16023 (2009). DOI: 10.1021/ja904138v

4. Dreef-Tromp CM, Lefeber AWM, van der Marel GA, van boom JH. Synthesis and phosphorylating properties of hydroxyamino acid phosphoramidites. *Synthesis* **12**, 1269-1272 (1992). DOI: 10.1055/s-1992-26355

5. Adamczyk M, Fishpaugh JR, Heuser KJ. Preparation of succinimidyl and pentafluorophenyl active esters of 5- and 6-carboxyfluorescein. *Bioconjug Chem* **8**, 253-255 (1997). DOI: 10.1021/bc9600877

6. Feng Z*, et al.* Ligand Depot: a data warehouse for ligands bound to macromolecules. *Bioinformatics* **20**, 2153-2155 (2004). DOI: 10.1903/bioinformatics/bth214

7. Kagami L, Wilter A, Diaz A, Vranken W. The ACPYPE web server for small-molecule MD topology generation. *Bioinformatics* **39**(6), btad350. (2023) DOI: 10.1093/bioinformatics/btad350

8. He X, Man VH, Yang W, Lee TS, Wang J. A fast and high-quality charge model for the next generation general AMBER force field. *J Chem. Phys.* **153**(11), 114502. (2020) DOI: 10.1063/5.0019056

9. Jumper, J., Evans, R., Pritzel, A. et al. Highly accurate protein structure prediction with AlphaFold. *Nature* **596**, 583–589. (2021) DOI: 10.1038/s41586-021-03819-2

10. Mihaly Varadi M, et al. AlphaFold Protein Structure Database in 2024: providing structure coverage for over 214 million protein sequences. *Nucleic Acids Res.* 2024, **52**(D1), D368–D375. (2024) DOI: 10.1093/nar/gkad1011

11. Gordon JC, Myers JB, Folta T, Shoja V, Heath LS, Onufriev A. H++: a server for estimating pKas and adding missing hydrogens to macromolecules. *Nucleic Acids Res.* **33** (Web Server issue), W368-71. (2005) DOI: 10.1093/nar/gki464

12. Lindorff-Larsen K, Piana S, Palmo K, Maragakis P, Klepeis JL, Dror RO, Shaw DE. Improved side-chain torsion potentials for the Amber ff99SB protein force field. *Proteins.* **78**(8), 1950-8 (2010) DOI: 10.1002/prot.22711

13. Abraham, M. J., Murtola, T., Schulz, R., Páll, S., Smith, J.C., Hess, B., et al. GROMACS: High performance molecular simulations through multi-level parallelism from laptops to supercomputers. *SoftwareX* **1**, 19–25 (2015) DOI: 10.1016/j.softx.2015.06.001

14. Jorgensen, W. L., Chandrasekhar, J., Madura, J. D., Impey, R. W., Klein, M. L. Comparison of simple potential functions for simulating liquid water. *J. Chem. Phys.* **79**(2), 926 (1998) DOI: 10.1063/1.445

15. Darden, T., York, D., Pedersen, L. Particle Mesh Ewald: An W Log(N) Method for Ewald sums in large systems. *J. Chem. Phys.* **98**, 10089 (1993) DOI: 10.1063/1.464397

16. Essmann, U., Perera, L., Berkowitz, M. L., Darden, T., Lee, H., & Pedersen, L. G. A smooth particle mesh Ewald method. *J. Chem. Phys.* **103**(19), 8577-8593 (1995). DOI: 10.1021/acs.jctc.6b00701

17. Berendsen, H. J. C., Postma, J. P. M., van Gunsteren, W. F., DiNola, A., Haak, J. R. Molecular dynamics with coupling to an external bath. *J. Chem. Phys.* **81**, 3684-3690. (1984) DOI: 10.1063/1.448118

18. Bussi, G., Donadio, D., Parrinello, M. Canonical sampling through velocity rescaling. *J. Chem. Phys.* **126**(1), 014101 (2007) DOI: 10.1063/1.2408420

19. Hess B. P-LINCS: A Parallel linear constraint solver for molecular simulation. *J. Chem. Theory Comput.* **4**(1), 116-22(2008) doi: 10.1021/ct700200b

20. Hess, B., Bekker, H., Berendsen, H. J. C., Fraaije, J. G. E. M. LINCS: A linear constraint solver for molecular simulations. *J. Comput. Chem.* **18**, 1463-1472 (1997)

21. Yan, Y., Yang, M., Ji, C. G., Zhang, J. Z. H. Interaction entropy for computational alanine scanning. *J. Chem. Inf. Model.*, **57**(5), 1112–1122 (2017) DOI: 10.1021/acs.jcim.6b0

22. Sun Z, Yan YN, Yang M, Zhang JZ. Interaction entropy for protein-protein binding. *J. Chem. Phys.* **146**(12), 124124 (2017) DOI: 10.1063/1.4978893

23. Knapp B, Ospina L, Deane CM. Avoiding false positive conclusions in molecular simulation: the importance of replicas. *J Chem Theory Comput.* **14**(12), 6127-6138 (2018). DOI: 10.1021/acs.jctc.8b00391.

24. Kumar S, Rosenberg JM, Bouzida D, Swendsen RH, Kollman PA. The weighted histogram analysis method for free-energy calculations on biomolecules. I. *The Method. J. Comput. Chem.* **13**(8), 1011-1021 (1992) DOI: 10.1002/jcc.540130812.

25. Valdés-Tresanco MS, Valdés-Tresanco ME, Valiente PA, Moreno E. gmx_MMPBSA: A new tool to perform end-state free energy calculations with GROMACS. *J. Chem. Theory Comput.* **17**(10), 6281-6291 (2021) DOI: 10.1021/acs.jctc.1c00645

26. Mishra SK, Koča J. Assessing the performance of MM/PBSA, MM/GBSA, and QM-MM/GBSA approaches on protein/carbohydrate complexes: effect of implicit solvent models, QM methods, and entropic contributions. *J. Phys. Chem. B*. **122**(34), 8113-8121 (2018) doi: 10.1021/acs.jpcb.8b03655

27. Gohlke, H., Kiel, C., Case, D. A. Insights into protein-protein binding by binding Free energy calculation and free energy decomposition for the Ras-Raf and Ras-RalGDS complexes. *J. Mol. Biol.* **330**(4), 891–913 (2003) DOI: 10.1016/S0022-2836(03)00610-7

28. Martins SA, Perez MA, Moreira IS, Sousa SF, Ramos MJ, Fernandes PA. Computational alanine scanning mutagenesis: MM-PBSA vs TI. *J. Chem. Theory Comput.* **9**(3), 1311-9 (2013) DOI: 10.1021/ct4000372

29. Yan, Y., Yang, M., Ji, C. G., Zhang, J. Z. H. Interaction entropy for computational alanine scanning. *J. Chem. Inf. Model.* **57**(5), 1112–1122 (2017) DOI: 10.1021/acs.jcim.6b00

30. Hou, T., Wang, J., Li, Y., Wang, W. Assessing the performance of the MM/PBSA and MM/GBSA Methods: 1. The accuracy of binding free energy calculations based on molecular dynamics simulations. *J. Chem. Inf. Model*. **51**, 69−82 (2011) doi: 10.1021/ci100275a.

31. Wang EC, Sun HY, Wang JM, Wang Z, Liu H, Zhang JZH, Hou TG. From End-Point Binding Free Energy Calculation with MM/PBSA and MM/ GBSA: Strategies and Applications in Drug Design. *Chem. Rev*. **119** (16), 9478–9508 (2019) doi: 10.1021/acs.chemrev.9b00055.

32. Abramson J*, et al.* Accurate structure prediction of biomolecular interactions with AlphaFold 3. *Nature* **630**, 493-500 (2024). DOI: 10.1038/s41586-024-07487-w

33. Zhou J*, et al.* Mechanism of phosphate sensing and signaling revealed by rice SPX1-PHR2 complex structure. *Nature Communications* **12**, 7040 (2021). DOI: 10.1038/s41467-021-27391-5

34. Baek M, McHugh R, Anishchenko I, Jiang H, Baker D, DiMaio F. Accurate prediction of protein-nucleic acid complexes using RoseTTAFoldNA. *Nat Methods* **21**, 117-121 (2024). DOI: 10.1038/s41592-023-02086-5
